# Inflammation‐Targeted Nanomedicines Alleviate Oxidative Stress and Reprogram Macrophages Polarization for Myocardial Infarction Treatment

**DOI:** 10.1002/advs.202308910

**Published:** 2024-04-06

**Authors:** Danrong Hu, Ran Li, Yicong Li, Meng Wang, Lu Wang, Shiqi Wang, Hongxin Cheng, Qing Zhang, Chenying Fu, Zhiyong Qian, Quan Wei

**Affiliations:** ^1^ Rehabilitation Medicine Center and Institute of Rehabilitation Medicine Key Laboratory of Rehabilitation Medicine in Sichuan Province State Key Laboratory of Biotherapy and Cancer Center West China Hospital Collaborative Innovation Center Sichuan University Chengdu Sichuan 610041 P. R. China; ^2^ National Clinical Research Center for Geriatrics Aging and Geriatric Mechanism Laboratory West China Hospital Sichuan University Chengdu Sichuan 610041 P. R. China

**Keywords:** inflammation, macrophage polarization, myocardial infarction, oxidative stress, targeted drug delivery

## Abstract

Myocardial infarction (MI) is a critical global health challenge, with current treatments limited by the complex MI microenvironment, particularly the excessive oxidative stress and intense inflammatory responses that exacerbate cardiac dysfunction and MI progression. Herein, a mannan‐based nanomedicine, Que@MOF/Man, is developed to target the inflammatory infarcted heart and deliver the antioxidative and anti‐inflammatory agent quercetin (Que), thereby facilitating a beneficial myocardial microenvironment for cardiac repair. The presence of mannan on the nanoparticle surface enables selective internalization by macrophages rather than cardiomyocytes. Que@MOF/Man effectively neutralizes reactive oxygen species in macrophages to reduce oxidative stress and promote their differentiation into a reparative phenotype, reconciling the inflammatory response and enhancing cardiomyocyte survival through intercellular communication. Owing to the recruitment of macrophages into inflamed myocardium post‐MI, in vivo, administration of Que@MOF/Man in MI rats revealed the specific distribution into the injured myocardium compared to free Que. Furthermore, Que@MOF/Man exhibited favorable results in resolving inflammation and protecting cardiomyocytes, thereby preventing further myocardial remodeling and improving cardiac function in MI rats. These findings collectively validate the rational design of an inflammation‐targeted delivery strategy to mitigate oxidative stress and modulate the inflammation response in the injured heart, presenting a therapeutic avenue for MI treatment.

## Graphical abstract

In article number 202308910, Danrong Hu, Ran Li, and co‐workers report the design, preparation, and evaluation of a newly constructed mannan‐based nanomedicine, Que@MOF/Man, which achieved inflammation‐targeting delivery of the antioxidative and anti‐inflammatory agent quercetin (Que) to the infarcted heart.

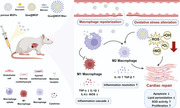

## 1 Introduction

Myocardial infarction (MI), a consequence of coronary artery occlusion, represents the most severe manifestation among all cardiovascular diseases.^[^ ^1^ ^]^ The reduction in blood flow to the injured heart initiates a series of microenvironmental changes, including ischemia, hypoxia, and the irreversible death of myocardial cells. Consequently, these events provoke a robust aseptic inflammatory response and an accumulation of reactive oxygen species (ROS), which significantly amplify the damage to the myocardial infarct zone.^[^ ^2^ ^]^ Timely reperfusion strategies, such as pharmacological thrombolysis and percutaneous coronary intervention, are the standard treatments for MI, effectively restoring oxygenated blood flow and salvaging ischemic myocardium from imminent cell death.^[^ ^3^ ^]^ However, these interventions only offer temporary symptomatic relief and fail to adequately support the long‐term maintenance of cardiac function.^[^ ^4^ ^]^ Furthermore, surgical interventions can intensify oxidative stress, trigger an inflammatory cascade, and cause additional myocardial injury during reperfusion, which contributes up to 50% of the final infarct size.^[^ ^5^ ^]^ Therefore, the identification of innovative therapeutic approaches is urgently needed for effective and targeted MI treatment, which holds potential clinical significance.

Similar to other types of tissue injury, macrophages play a pivotal role in modulating the immune response throughout the various stages of MI, including the inflammatory, proliferative (anti‐inflammatory), and reparative phases.^[^ ^6^ ^]^ Macrophages can be categorized into 2 distinct phenotypes based on their activation state: the pro‐inflammatory M1 phenotype and the anti‐inflammatory M2 phenotype.^[^ ^7^ ^]^ In the early stages following MI, innate and adaptive immune cells, particularly macrophages, are rapidly recruited to the infarcted area. These macrophages contribute to a pro‐inflammatory response, facilitating efferocytosis and the clearance of damaged tissue.^[^ ^8^ ^]^ The peak recruitment of M1 macrophages typically occurs around 3 days post‐MI, characterizing the inflammatory phase.^[^ ^9^ ^]^ Subsequently, anti‐inflammatory M2 macrophages become increasingly prevalent in the later stages of MI, secreting cytokines and growth factors that promote healing and remodeling of cardiac tissues.^[^ ^10^ ^]^ Appropriate inflammatory response plays a positive role in improving cardiac function after MI, which can not only effectively clear the dead cells in the infarction area, but also promote angiogenesis and the repair of cardiac structure^[^ ^11^ ^]^. However, beyond the duration, the intensity of macrophage‐mediated inflammatory response is closely associated with myocardial injury and scar formation.^[^ ^12^ ^]^ To be specific, excessive early inflammation predicts poor clinical outcomes for recovery.^[^ ^13^ ^]^ M1 macrophages in the injured heart play a critical role in the inflammatory cascade by continuously secreting pro‐inflammatory cytokines such as tumor necrosis factor‐alpha (TNF‐α), interleukin‐1β (IL‐1β), and interleukin‐6 (IL‐6), which recruit additional macrophages and immune cells from the circulation. They also produce ROS that disrupts redox homeostasis, leading to oxidative stress.^[^ ^14^ ^]^ Importantly, ROS within the MI microenvironments acts as critical signaling molecules, guiding the differentiation of recruited macrophages toward a pro‐inflammatory M1 phenotype rather than an anti‐inflammatory M2 phenotype, thereby intensifying the inflammatory response and exacerbating heart injury.^[^ ^15^ ^]^ Therefore, excessive inflammation and ROS overproduction by macrophages can a vicious cycle that, if left unchecked, leads to sustained damage and improper healing after MI.^[^ ^16^ ^]^ Therefore, it is essential to intervene promptly to disrupt this cycle to improve the pathological microenvironment post‐MI and ensure effective cardiac repair. Concurrently, pharmacological strategies that modulate the transition from a pro‐inflammatory to an anti‐inflammatory state have shown the potential to significantly improve long‐term cardiac repair outcomes, presenting a promising approach for MI treatment.^[^ ^17^ ^]^

Quercetin (Que), a natural polyphenol flavonoid recognized by the FDA as a Generally Recognized As Safe (GRAS) ingredient, holds substantial therapeutic potential for the treatment of various cardiovascular diseases owing to its potent antioxidant and anti‐inflammatory properties.^[^ ^18^ ^]^ The antioxidant activity of Que is attributed to its ability to scavenge ROS, a process facilitated by its intrinsic antioxidant pharmacophores, such as the catechol group, the 2,3‐double bond, and the hydroxyl group in its heterocyclic ring.^[^ ^19^ ^]^ Que has emerged as a cardioprotective agent against cardiac ischemia/reperfusion injury by inhibiting the inflammatory cascade and apoptosis through multiple signaling pathways, including PI3K/Akt pathway.^[^ ^20^ ^]^ Moreover, research suggests that the cardioprotective effect of Que is mediated by its ability to mitigate the release of inflammatory mediators and modulate the nuclear factor‐kappa B (NF‐κB) signaling pathway in macrophages, thereby influencing macrophage‐cardiomyocytes crosstalk.^[^ ^21^ ^]^ Que has also been shown to alleviate inflammation in other acute and chronic conditions, such as acute kidney injury,^[^ ^22^ ^]^ diabetes,^[^ ^23^ ^]^ and volumetric muscle loss injury^[^ ^24^ ^]^ by suppressing M1 phenotype polarization. Notably, Que has also been demonstrated to promote M2 phenotype polarization, creating a pro‐chondrogenic microenvironment for cartilage regeneration ^[^ ^25^ ^]^ and promoting periodontal tissue regeneration promotion.^[^ ^26^ ^]^ As a ROS scavenger and regulator of macrophage polarization, Que exhibits significant promise in treating MI by inhibiting post‐MI inflammation while specifically targeting recruited macrophages. Despite its remarkable bioactivities, Que's application is significantly constrained by its unfavorable physicochemical and pharmacokinetic characteristics, notably its poor water solubility. Several delivery systems have been developed to enhance the Que's bioavailability, but most focus on traditionally local delivery, lacking active targeting capabilities for inflammatory lesions. Given the significance of inhibiting excessive inflammatory responses and scavenging ROS in macrophages for the survival of cardiomyocytes, it is crucial to effectively deliver Que to the recruited macrophages in the inflamed myocardium and provide sustained cardiomyocyte protection through intercellular communication.

**Figure 1:**
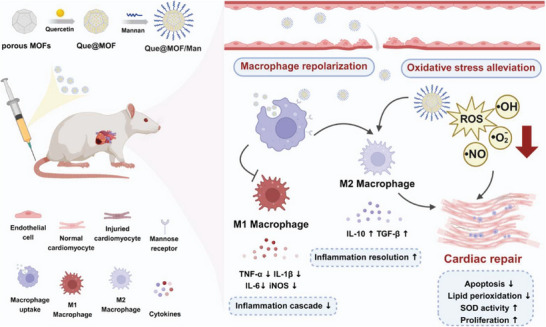
Schematic illustration of the inflammation‐targeting Que@MOF/Man nanoparticles for antioxidant and anti‐inflammatory treatment against MI injury.

To improve the therapeutic efficacy of MI, we developed inflammation‐targeted nanoparticles, Que@MOF/Man, designed for the selective delivery of Que to injured myocardium and recruited macrophages in a controlled manner. As a proof of concept, a mannan (Man)‐functionalized nano‐metal‐organic framework (MOF) was fabricated to encapsulate Que for site‐directed therapy of MI. Zeolitic imidazolate framework 8 (ZIF‐8) nanoparticles, a widely studied MOF for biomedical purposes, offer a large internal space for drug encapsulation and the ability to release drugs on‐demand in the acidic microenvironment of MI due to their acidic decomposition properties.^[^ ^27^ ^]^ The surface modification of ZIF‐8 nanoparticles with negatively charged Man via electrostatic interaction is expected to enhance the safety and in vivo stability of Que@MOF/Man nanoparticles. More importantly, Man, a yeast polysaccharide containing D‐mannose residues, is recognized by mannose receptors, which are highly expressed on antigen‐presenting cells, particularly macrophages at sites of inflammation.^[^ ^28^ ^]^ This recognition facilitates inflammation‐specific accumulation and targeted cellular internalization of Que@MOF/Man in macrophages. Following intravenous administration, Que@MOF/Man is expected to accumulate at the injured myocardium due to the enhanced permeability and retention (EPR) effect induced by MI.^[^ ^29^ ^]^ In the infarcted area, where inflammatory macrophages are abundant, the Man on the surface of Que@MOF/Man nanoparticles can specifically recognize and bind to the carbohydrate recognition domains of mannose receptors on macrophages, promoting the endocytosis of the nanoparticles. The intracellularly delivered Que is expected to alleviate oxidative stress in inflammatory macrophages, induce an anti‐inflammatory phenotype, and exert cardioprotective effects (**Figure** **1**, Scheme). Consequently, Que@MOF/Man nanoparticles serve as a “magic bullet”, precisely targeting inflamed sites to achieve antioxidant and anti‐inflammatory therapy, thereby promoting the cardiac repair of MI.

## 2 Results and Discussion

### 2.1 Synthesis and Characterization of Que@MOF/Man

**Figure 2:**
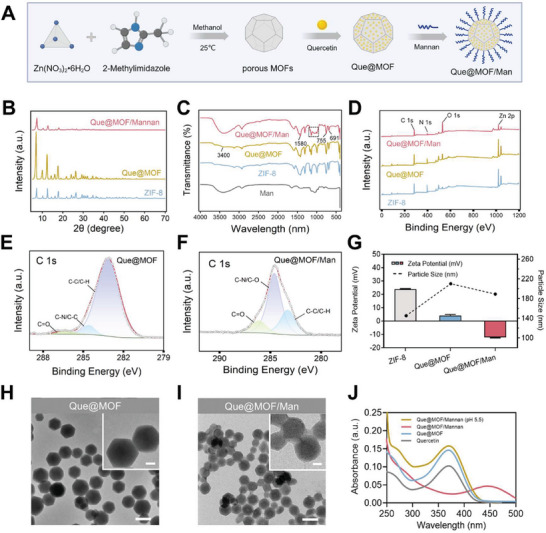
Preparation and characterization of Que@MOF/Man. A) Schematic illustration of the synthesis routine. B) XRD patterns of Que@MOF/Man, Que@MOF, and ZIF‐8. C) FTIR spectra of Que@MOF/Man, Que@MOF, ZIF‐8, and Man. D) XPS full spectra of Que@MOF/Man, Que@MOF, and ZIF‐8. E,F) XPS C 1s spectrum of Que@MOF (E) and Que@MOF/Man (F). G) Zeta potential and particle size for Que@MOF/Man, Que@MOF, and ZIF‐8 measured using dynamic light scattering (DLS). H,I) TEM images of Que@MOF (H) and Que@MOF/Man (I). Scale bar, 200 and 50 nm (insert). J) UV–Vis spectra of Que@MOF/Man (pH 5.5), Que@MOF/Man, Que@MOF, and Que.

As depicted in **Figure** **2**A, the synthesis of Que@MOF/Man nanoparticles is a three‐step process. First, ZIF‐8 nanoparticles are produced using a one‐step method, employing dimethylimidazole as the organic ligand and zinc nitrate as the zinc source. Next, hydrophobic Que is encapsulated within the nanoparticle core via coordination with zinc ions, resulting in Que@MOF. Finally, Man is introduced to the surface of Que@MOF via electrostatic adsorption to form Que@MOF/Man nanoparticles. The X‐ray diffraction (XRD) pattern showed that the Que@MOF and Que@MOF/Man shared characteristic diffraction peaks with ZIF‐8 nanoparticles (Figure 2B), indicating that the encapsulation of Que and the coating of Man do not compromise the crystalline structure of ZIF‐8. Notably, the peak intensity of Que@MOF is stronger compared to ZIF‐8, suggesting that Que may influence the crystallinity of ZIF‐8. Nevertheless, the peak intensity in Que@MOF/Man is diminished, likely due to the Man coating affecting the diffraction efficiency.

Furthermore, the chemical composition of Que@MOF was further confirmed by the Fourier‐transform infrared (FT‐IR) spectra (Figure 2C). The characteristic peaks of ZIF‐8, including the C═N stretching vibration peak at 1580 cm^−1^, the Zn‐O coordination bonding at 755 cm^−1^ and the Zn‐N coordination bonding at 691 cm^−1^, were observed in both Que@MOF and Que@MOF/Man. The FT‐IR spectra of Que@MOF exhibited a significantly weakened O‐H bonding absorption peak at 3400 cm^−1^, indicating that the hydroxyl‐rich Que is predominantly encapsulated within ZIF‐8 pores. The FT‐IR spectra of Que@MOF/Man exhibited a distinctive “fingerprint” region between 1000 and 1200 cm^−1^, which was attributed to the stretching vibration of carbohydrate bonds, confirming the successful surface modification of Que@MOF with Man.^[^ ^30^ ^]^ X‐ray photoelectron spectroscopy (XPS) analysis further validated the successful synthesis of the composite nanoparticles, as evidenced by the presence of the Zn 2p peak in the full scan spectrum of Que@MOF and Que@MOF/Man (Figure 2D). In terms of C 1s spectrum, the appearance of the C═O at 286.3 eV peak in Que@MOF/Man indicated the successful encapsulation of Que, in contrast to the absence of this peak in ZIF‐8 (Figure 2E; Figure S1, Supporting Information). Furthermore, the post‐modification with Man led to a significant enhancement in the peak intensity of C═O at 286.0 eV and C‐N/C‐O at 284.6 eV, aligning with the aforementioned observations (Figure 2F).

The N_2_ adsorption‐desorption isotherms were used to determine the porosity of nanoparticles (Figure S2, Supporting Information). The specific surface areas of ZIF‐8, Que@MOF and Que@MOF/Man were determined to be 1889.57, 1598.77, and 3.21 m^2^ g^−1^, respectively, using the Brunauer‐Emmett‐Teller (BET) mode. A slight decrease in the specific surface area of Que@MOF compared to ZIF‐8 suggests the incorporation of Que within the ZIF‐8 pores, while a more significant reduction in Que@MOF/Man indicates that the Man coating largely masks the nanoparticle pores.

Transmission electron microscopy (TEM) images revealed that both Que@MOF and Que@MOF/Man had smoother and more spherical surfaces than the regular dodecahedral‐shaped ZIF‐8, with Que@MOF/Man exhibiting a pronounced change in morphology (Figure 2H; Figure S3, Supporting Information). Dynamic light scattering (DLS) analysis showed that the average particle sizes of ZIF‐8, Que@MOF, and Que@MOF/Man were 145.6, 210.0, and 189.9 nm, respectively (Figure 2G). Moreover, the zeta potential of Que@MOF/Man registered at ‐12.17 mV, compared to 23.8 mV for ZIF‐8 and 4.06 mV for Que@MOF (Figure 2G), suggesting enhanced biocompatibility and stability due to the hydroxyl groups in Man, which reduce protein adsorption during blood circulation. Recognizing the critical importance of nanoparticle stability for biomedical applications, we conducted a DLS assay to evaluate the aqueous stability of both unmodified ZIF‐8 and Que@MOF/Man nanoparticles. Our results revealed that bare ZIF‐8 exhibited limited solution stability, maintaining its original state for a maximum of 24 h. Moreover, a notable increase in particle size, exceeding 1000 nm, was observed within 7 days, indicative of structural degradation (Figure S4, Supporting Information). In contrast, Que@MOF/Man exhibited negligible changes in particle size over the same time period, with only a slight increase in the polydispersity index (PDI), thereby confirming their superior stability compared to ZIF‐8.

The drug loading content of Que was determined using UV–Vis spectrophotometry, with a value of approximately 5.2% based on its characteristic absorption peak at 374 nm. Interestingly, there was a distinct shift in the Que absorption from 374 to 447 nm after Man modification (Figure 2J), which can be attributed to the surface coating concealing Que within the nanoparticles. Nonetheless, when the pH was adjusted to 5.5, the absorption peak shape of Que@MOF/Man returned to a feature closely resembling that of free Que, potentially related to the acidic degradation of ZIF‐8, which facilitates drug release.^[^ ^31^ ^]^

### 2.2 Free Radicals Scavenging Ability In Vitro

**Figure 3:**
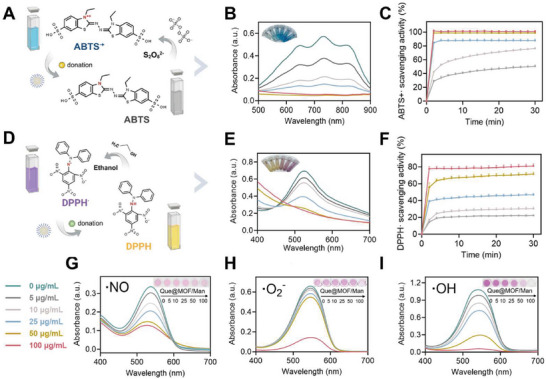
Free radical scavenging capability of Que@MOF/Man. A,D) Schematic representation of scavenging capacity of Que@MOF/Man for ABTS^•+^ (A) and DPPH^•^ (D). B,E) UV–Vis spectra of ABTS^•+^ (B) and DPPH^•^ (E) exposure to different concentrations of Que@MOF/Man. Inset: the digital photographs showing the color of each reaction solution. C,F) Kinetic curves of the scavenging capacity of Que@MOF/Man at different concentrations for ABTS^•+^ (C) and DPPH^•^ (F) in PBS (pH 5.5). G–I) UV−Vis spectra showing the radicals eliminating activities of Que@MOF/Man for •NO (G), •O_2_^−^ (H), and •OH (I). Inset: the digital photographs showing the color of each reaction solution.

Que, renowned for its potent antioxidant properties, has attracted considerable attention as a natural radical scavenger.^[^ ^32^ ^]^ We thus evaluated the antioxidant capacity of Que@MOF/Man against various free radicals under aqueous conditions. First, the efficacy of Que@MOF/Man was assessed at different concentrations (5, 10, 25, 50, 100 µg mL^−1^) using the standard free‐radical reagents ABTS^•+^ and DPPH^•^ in PBS at pH 5.5, with a total incubation time of 30 min. This experiment was designed to mimic the acidic ischemic microenvironment typically encountered in vivo.^[^ ^33^ ^]^ The ABTS^•+^ radicals were generated by oxidizing ABTS with potassium persulfate, which exhibits a blue color and characteristic UV‐vis absorption peak at 734 nm (**Figure** **3**A).^[^ ^34^ ^]^ Que@MOF/Man exhibited a concentration‐dependent manner in quenching ABTS^•+^, with a corresponding color change from blue to colorless in the solution (Figure 3B). The radical scavenging kinetics and rate were also concentration‐ and time‐dependent, with Que@MOF/Man (100 µg mL^−1^) achieving an ABTS^•+^ scavenging ratio of 98.6% after a 30‐min incubation (Figure 3C). The DPPH^•^ scavenging activity of Que@MOF/Man was further investigated, with the DPPH^•^ ethanol solution showing a dark purple color and an absorption peak at 517 nm (Figure 3D).^[^ ^35^ ^]^ The absorption peak consistently decreased with increasing concentrations of Que@MOF/Man in DPPH^•^ solution, transitioning from purple to yellow (Figure 3E). The scavenging efficiency of Que@MOF/Man against DPPH^•^ was concentration‐ and time‐dependent, reaching a scavenging rate of 81.3% at a concentration of 100 µg mL^−1^ after 30 min‐incubation (Figure 3F). The ABTS^•+^ and DPPH^•^ scavenging properties of Que@MOF/Man were also assessed in PBS 7.4 to mimic the normal physiological conditions (Figure S5, Supporting Information). At a 30‐min incubation time, the scavenging percentages of Que@MOF/Man (100 µg mL^−1^) in PBS 7.4 for ABTS^•+^ and DPPH^•^ were 94.6% and 74.7%, respectively, which may be attributed to the inadequate degradation of ZIF‐8 under these conditions.

Subsequently, we comprehensively evaluated the antioxidant capacity of Que@MOF/Man against a range of physiologically relevant radicals, including nitric oxide radical (•NO), superoxide anion radical (•O_2_^−^), and hydroxyl radical (•OH). The •NO radical, recognized as a ROS, can react with •O_2_^−^ to generate ONOO^−^, a potent oxidant and nitrating agent associated with significant oxidative and nitrating stress.^[^ ^36^ ^]^ The •NO radical scavenging capacity of Que@MOF/Man was assessed using Griess reagents to detect nitrite and nitrate as oxidation products. With increasing concentrations of Que@MOF/Man, a progressive decrease in the absorbance peak at 540 nm was observed, indicating a high antioxidant performance with a concentration‐dependent pattern (Figure 3G). The •O_2_^−^ radical, a primary ROS generated as a byproduct in the mitochondrial electron transport chain, was evaluated for its scavenging ability using a superoxide dismutase (SOD)‐like activity with a xanthine and xanthine oxidase reaction system. Que@MOF/Man demonstrated a concentration‐dependent scavenging effect on •O_2_^−^ radicals, as evidenced by the gradual fading of the color (Figure 3H). The •OH radical, known for its strong oxidizing capacity among ROS, is highly cytotoxic, causing DNA damage and lipid peroxidation.^[^ ^37^ ^]^ With the increased concentration of Que@MOF/Man, a decrease in characteristic absorbance and a color change from pink to colorless in the Fenton reaction solution was observed, indicating the effective elimination of the generated •OH radicals by Que@MOF/Man (Figure 3I).

### 2.3 In Vitro Biocompatibility

The biocompatibility of Que@MOF/Man is a fundamental requirement for its biomedical applications, particularly for therapeutic purposes. Given that these nanoparticles are designed to circulate directly within the bloodstream, we initially investigated their hemolytic potential by incubating rat erythrocytes with different concentrations of Que@MOF/Man and ZIF‐8. The results indicated that Que@MOF/Man exhibited a lower hemolysis rate compared to ZIF‐8 at equivalent concentrations (Figure S6, Supporting Information). In comparison to the positive control group, negligible hemolysis (< 5%) was observed across all groups, demonstrating good blood compatibility. Subsequently, we evaluated the cytobiocompatibility of Que@MOF/Man on RAW 264.7 macrophages and H9C2 cardiomyocytes using CCK8 assay. The results revealed that Que@MOF/Man maintained cell viability at up to 80% even at a high concentration of 100 µg mL^−1^ (Figure S7A,B, Supporting Information). Notably, the cell viabilities of H9C2 and RAW 264.7 cells treated with Que@MOF/Man at ZIF‐8 concentrations of 75 and 100 µg mL^−1^ were significantly higher than those of corresponding cells treated with ZIF‐8 (Figure S7C,D, Supporting Information). These remarkable biocompatible characteristics offer assurance for further investigation into its biological function and application in the treatment of MI.

### 2.4 Specific Macrophage‐Targeting and Selective Cellular Uptake

Macrophages play a critical role in the inflammatory response and tissue repair post‐MI, while cardiomyocytes are essential for maintaining cardiac function, making both cell types vital for MI treatment.^[^ ^9^, ^38^ ^]^ Given the excessive ROS and inflammatory cascade triggered by macrophages post‐MI, targeted delivery of Que to macrophages in the infarcted heart is beneficial for its anti‐oxidative and anti‐inflammatory effects. Additionally, the survival of cardiomyocytes post‐MI is crucial for improving heart function. It is thus pivotal to assess whether nanoparticles exert therapeutic effects on cardiomyocytes either directly through endocytosis or indirectly via interactions with immune cells, such as macrophages.

**Figure 4:**
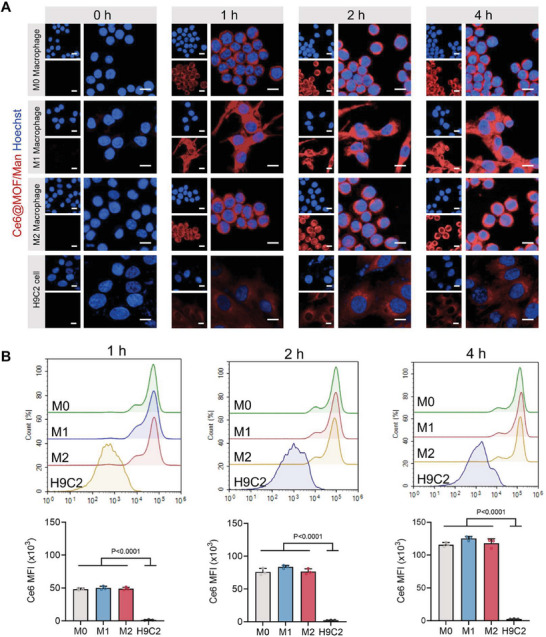
In vitro targeting ability and macrophage‐specific cellular uptake of Ce6@MOF/Man. A) Representative confocal images of RAW 264.7 macrophages and H9C2 cardiomyocytes treated with Ce6@MOF/Man (red) at various time points (0, 1, 2, and 4 h). Nuclei were labeled with Hoechst 33342. Scale bar, 20 µm. (B) Quantitative analysis for intracellular Ce6@MOF/Man uptake in RAW 264.7 macrophages and H9C2 cardiomyocytes over different incubation periods (1, 2, and 4 h) as assessed by flow cytometry. Mean fluorescence intensity (MFI) values are presented as mean ± SD (*n* = 3).

To this end, we initially investigated the phagocytic capabilities of macrophages and cardiomyocytes for nanoparticles. Herein, a fluorescence probe, chlorin e6 (Ce6), was encapsulated in Ce6@MOF/Man to visually track the cellular uptake process. The particle size and zeta potential of Ce6@MOF/Man were confirmed to be similar to those of Que@MOF/Man (Figure S8, Supporting Information). The intracellular uptake of Ce6@MOF/Man by RAW 264.7 cells and H9C2 cells at different time points (1, 2, and 4 h) was evaluated using confocal laser scanning microscopy (CLSM) and flow cytometry. Macrophages were subjected to a simulated inflammatory microenvironment, including naive macrophages (M0), pro‐inflammatory macrophages induced by LPS (M1), and anti‐inflammatory macrophages induced by IL‐4 and IL‐13 (M2). Fluorescent images showed that Ce6@MOF/Man (red) were extensively internalized within the cytoplasm of various macrophages, whether M0, M1, or M2 macrophages, while minimal fluorescence signals were observed in cardiomyocytes following 1, 2, and 4 h‐incubation (**Figure** **4**A).

The quantification result of cellular uptake by flow cytometry further confirmed the preferential uptake of Ce6@MOF/Man by macrophages, which was at least 30‐fold higher than that in cardiomyocytes (Figure 4B). Mannan‐based Que@MOF/Man nanoparticles are supposed to be preferentially internalized by M2 macrophages through their binding affinity with the upregulated CD206 receptor on the macrophage surface. Nevertheless, fluorescence quantification analysis using CLSM and flow cytometry revealed that M2 macrophages did not exhibit superior phagocytosis of nanoparticles compared to other macrophage subtypes (Figure 4A; Figure S9, Supporting Information), indicating that all macrophage subtypes have a similar capacity for cellular uptake. This observation suggests that the nanoparticles we have engineered possess the capability to efficiently deliver Que to the inflammatory macrophages. Furthermore, the intracellular uptake of Ce6@MOF/Man by macrophages occurred in a time‐dependent manner, as per the flow cytometry quantitative analysis, while cardiomyocytes showed a limited enhancement in internalizing nanoparticles with increasing incubation time (Figure S9, Supporting Information). These findings underscore the significant targeting capacity of inflammatory sites and the specific uptake by macrophages, aligning with our design intent. Given the significant recruitment of macrophages in the injured myocardium post‐MI, Que@MOF/Man emerges as a promising candidate for an actively targeted therapeutic approach in MI.

### 2.5 Anti‐Oxidative and Anti‐Inflammatory Ability In Vitro

**Figure 5:**
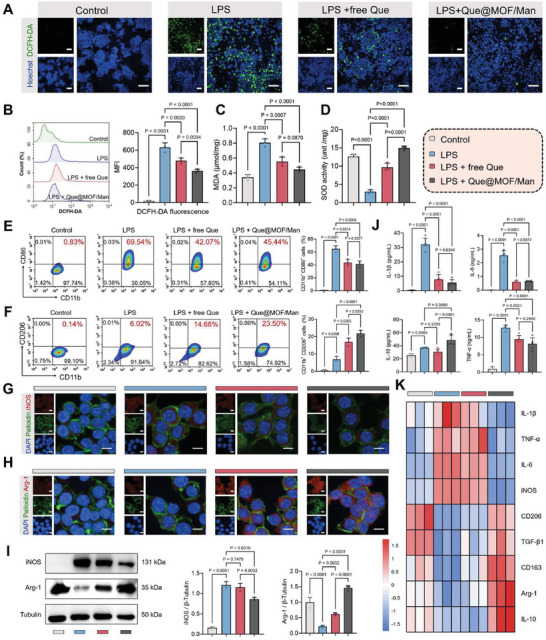
In vitro antioxidant and anti‐inflammatory effects on macrophages. A,B) Representative confocal images (A) and flow cytometry analysis (B) for ROS production in RAW 264.7 cells following different treatments, with DCFH‐DA (green) as an indicator (*n* = 3). Scale bar, 100 µm. C,D) Quantification of malondialdehyde (MDA) level (C) and superoxide dismutase (SOD) activity (D) in RAW 264.7 cells after different treatments (*n* = 3). E,F) Flow cytometric analysis for the proportion of M1 phenotype (CD86^+^ CD11b^+^) and M2 phenotype (CD206^+^ CD11b^+^) in RAW 264.7 cells after different treatments (*n* = 3). G,H) Representative confocal images of M1 marker (iNOS) and M2 marker (Arg‐1) expression in RAW 264.7 cells after different treatments, as determined by immunofluorescence staining. Scale bar, 10 µm. I) Western blot analysis and quantification of iNOS and Arg‐1 expression in RAW 264.7 cells after different treatments (*n* = 3). J) ELISA‐based quantification of pro‐inflammatory cytokines (IL‐1β, IL‐6, and TNF‐α) and anti‐inflammatory cytokine (IL‐10) secretion in RAW 264.7 cells after different treatments (*n* = 5). K) Relative gene expression levels of pro‐inflammatory (IL‐1β, TNF‐α, IL‐6, and iNOS) and anti‐inflammatory (CD206, TGF‐β1, CD163, Arg‐1, and IL‐10) cytokines and molecules in RAW 264.7 cells after different treatments, as assessed by RT‐qPCR assay (*n* = 3). Data are presented as mean ± SD.

Inspired by the outstanding free radical scavenging properties and macrophage‐targeting capabilities of Que@MOF/Man, we initially assessed its intracellular ROS scavenging efficacy in macrophages using the DCFH‐DA probe, employing both confocal laser scanning microscopy (CLSM) and flow cytometry. LPS was first employed to induce inflammation‐associated oxidative stress in RAW 264.7 cells. As depicted in **Figure** **5**A, a pronounced green fluorescence was observed in LPS‐activated macrophages, indicating an obvious production of intracellular ROS due to the excessive inflammation triggered by LPS. Following treatment with free Que and Que@MOF/Man, a remarkable reduction in green fluorescence was observed in both groups. Notably, Que@MOF/Man exerted a more pronounced reduction in green fluorescence compared to free Que, indicating the superior intracellular ROS scavenging performance. This phenomenon might be attributed to the enhanced uptake of Que@MOF/Man through Man‐mediated endocytosis, resulting in elevated intracellular Que levels. Flow cytometry assay further confirmed that the proportion of ROS‐positive macrophages induced by LPS was significantly reduced in Que@MOF/Man (42.79% ± 2.75%) and free Que (24.09% ± 4.01%) groups, with Que@MOF/Man showing the most striking ROS scavenging ability (*p *< 0.0001, Figure 5B).

Excessive ROS generation can induce membrane lipid peroxidation and negatively affect anti‐oxidative enzyme activities, resulting in increased malondialdehyde (MDA) levels^[^ ^39^ ^]^ and decreased superoxide dismutase (SOD) activity.^[^ ^40^ ^]^ Therefore, MDA content and SOD activity were then investigated in RAW 264.7 cells. LPS‐stimulated macrophages exhibited a notable increase in MDA content from 0.34 to 0.81 µmol mg^−1^ and a significant decrease in SOD activity from 12.62 to 3.01 unit mg^−1^. However, treatment with free Que and Que@MOF/Man significantly mitigated MDA accumulation and enhanced SOD activity in pro‐inflammatory cells (Figure 5C,D). Collectively, Que@MOF/Man can effectively alleviate inflammation‐induced oxidative stress in macrophages.

The interplay between oxidative stress and inflammatory response is pivotal in cardiac repair following MI.^[^ ^41^ ^]^ Under normal physiological conditions, a balance is maintained between pro‐inflammatory M1 macrophages and anti‐inflammatory M2 macrophages. However, the induction of oxidative stress and heightened inflammation disrupt this homeostasis, leading to a predominance of pro‐inflammatory M1 macrophages.^[^ ^42^ ^]^ Consequently, it is imperative to modulate the activation of regenerative M2 macrophages to suppress inflammation progression. LPS was initially used to induce an inflammatory activation, followed by the addition of free Que and Que@MOF/Man to pro‐inflammatory RAW 264.7 cells. Unstimulated cells served as a control. Flow cytometry was employed to directly assess the interaction of Que@MOF/Man with macrophage phenotypes by detecting the specific markers for the M1 phenotype (CD86) and the M2 phenotype (CD206). The findings revealed that both Que@MOF/Man and free Que similarly suppressed the M1 macrophage phenotype, with a reduction in the inflammatory marker CD86 expression to 45.44% and 42.07%, respectively, in comparison to the untreated LPS‐induced group, which exhibited a CD86 expression level of 69.54% (Figure 5E). Conversely, the Que@MOF/Man group exhibited a higher proportion of CD206^+^ macrophages (23.50%), demonstrating superior facilitation of the M2 phenotype, while the free Que group showed a smaller increase (14.66%) compared to the LPS‐induced group (6.02%) (Figure 5F).

For a more in‐depth investigation of inflammation regulation within the MI microenvironment, RAW 264.7 cells were subjected to immunofluorescence staining with iNOS (a specific marker for M1 phenotype) and Arg‐1 (a specific marker for M2 phenotype). Que@MOF/Man treatment resulted in a noticeable decrease in iNOS expression and a significant increase in Arg‐1 expression compared to LPS‐induced group (Figure 5G,H). Western‐blot analysis confirmed that macrophages treated with Que@MOF/Man exhibited reduced iNOS expression and elevated Arg‐1 expression, which was consistent with the immunofluorescence staining results (Figure 5I). These findings suggest that Que@MOF/Man can effectively attenuate pro‐inflammatory differentiation induced by LPS and enhance the activation of a regenerative macrophage phenotype.

Subsequently, the levels of typical cytokines were evaluated. As depicted in Figure 5J, Que@MOF/Man treatment was found to inhibit the secretion of pro‐inflammatory cytokines, such as IL‐1β, IL‐6, and TNF‐α, while enhancing the secretion levels of anti‐inflammatory cytokine IL‐10 in macrophages, as determined through enzyme‐linked immunosorbent assay (ELISA). The expression of genes associated with pro‐inflammatory and anti‐inflammatory factors was then evaluated by real‐time quantitative (RT‐qPCR). The heat map of gene expression revealed that Que@MOF/Man significantly down‐regulated genes associated with pro‐inflammatory cytokines and molecules, such as IL‐1β, TNF‐α, IL‐6, and iNOS, and up‐regulated the expression of genes associated with anti‐inflammatory cytokines and molecules, such as CD206, TGF‐β1, CD163, Arg‐1, and IL‐10, compared with the LPS‐induced group (Figure 5K). These results revealed that Que@MOF/Man can effectively alleviate inflammation‐induced oxidative stress and facilitate macrophage differentiation toward a reparative phenotype within the challenging context of an oxidative and inflammatory microenvironment. This, in turn, serves to inhibit the inflammatory cascade and enhance the inflammation resolution.

### 2.6 Macrophage‐Mediated Protective Effects on Cardiomyocytes

**Figure 6:**
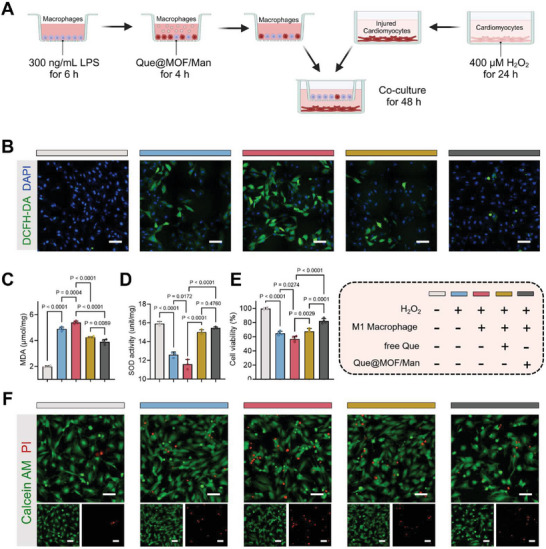
Cardioprotective effects mediated by Que@MOF/Man‐treated macrophages. A) Schematic illustration of the co‐culture assay in vitro. B) Representative images of ROS production in cardiomyocytes co‐cultured with macrophages from each group, as detected by DCFH‐DA staining. Scale bar, 50 µm. (C‐E) Quantification analysis for malondialdehyde (MDA) content (*n* = 4) C), superoxide dismutase (SOD) activity (*n* = 3) D), and cell viability (*n* = 4) (E) of cardiomyocytes co‐cultured with macrophages from each group. F) Representative Live/Dead staining images of cardiomyocytes co‐cultured with macrophages from each group. Scale bar, 50 µm. Data are presented as mean ± SD.

MI injury triggers cardiomyocyte necrosis and apoptosis, which in turn prompts the recruitment of macrophages and initiates an inflammatory cascade. These changes in the intricate microenvironment further exacerbate the progression and deterioration of MI. The aforementioned results have highlighted the beneficial effects of Que@MOF/Man on inflammatory macrophages. Considering the pivotal role of macrophage‐cardiomyocyte communication in the progression of cardiovascular disease,^[^ ^43^ ^]^ we thus investigated the effect of Que@MOF/Man‐treated macrophages on cardiomyocytes. To mimic the MI microenvironment in vitro, LPS‐induced RAW 264.7 cells and H_2_O_2_‐induced H9C2 cells were co‐cultured (**Figure** **6**A). The macrophage‐mediated ROS scavenging effect on injured cardiomyocytes was initially assessed using CLSM with DCFH‐DA a probe. As depicted in Figure 6B, H_2_O_2_ treatment led to a significant increase in ROS production by cardiomyocytes, and co‐culturing with LPS‐treated macrophages further intensified ROS accumulation. Nevertheless, co‐culturing with Que@MOF/Man‐treated macrophages appeared to inhibit ROS production in the injured cardiomyocytes (Figure 6B). Furthermore, co‐culturing with Que@MOF/Man‐treated macrophages demonstrated a notable protective effect against oxidative stress in H_2_O_2_‐injured cardiomyocytes, as evidenced by a 27.90% decrease in MDA content (Figure 6C) and a 33.61% increase in SOD activity (Figure 6D), when compared to H_2_O_2_‐injured cardiomyocytes co‐cultured with macrophages induced by LPS. These findings implied that macrophage differentiation induced by Que@MOF/Man effectively mitigates oxidative stress in damaged cardiomyocytes through intercellular communication and interaction. Additionally, H_2_O_2_‐treated cardiomyocytes exhibited a significant increase in cell death, with only 64.81% remaining viable. Co‐culturing these cardiomyocytes with LPS‐induced macrophages further intensified cell death, reducing cell viability to 56.91%. However, co‐culturing with macrophages treated with Que@MOF/Man alleviated cardiomyocyte apoptosis under oxidative and inflammatory conditions, as indicated by an increase in cell viability to 82.09% (Figure 6E). Notably, live/dead fluorescent staining of cardiomyocytes co‐cultured with Que@MOF/Man‐treated macrophages showed a predominance of live cells (green) with minimal dead cells (red) detected (Figure 6F). These results indicated that Que@MOF/Man‐treated macrophages can protect cardiomyocytes from oxidative stress and inflammation‐induced injury.

### 2.7 Transcriptomic Analysis of Molecular Mechanism

**Figure 7:**
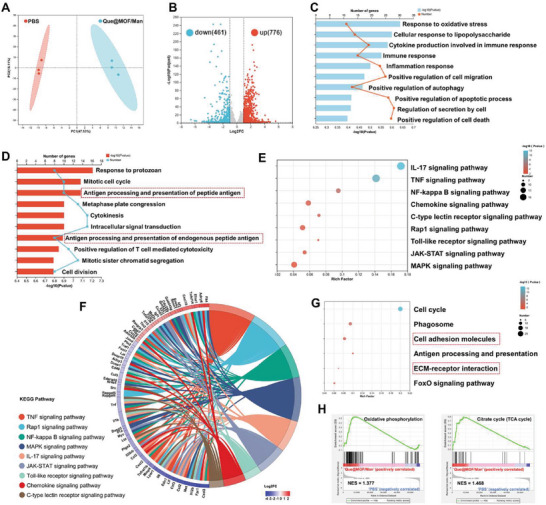
Transcriptome analysis for the effects of Que@MOF/Man on LPS‐induced RAW 264.7 cells. A) Principal component analysis (PCA) of gene expression profiles in LPS‐induced RAW 264.7 cells following PBS and Que@MOF/Man treatments. Each point describes each sample (*n* = 3). B) Volcano plot of differential expression genes (DEGs) between PBS and Que@MOF/Man group. C,D) Gene Ontology (GO) enrichment analysis for down‐regulated (C) and up‐regulated (D) DEGs between PBS and Que@MOF/Man group. E,G) Kyoto Encyclopedia of Genes and Genomes (KEGG) pathway enrichment analysis for down‐regulated (E) and up‐regulated (G) DEGs between PBS and Que@MOF/Man group. (F) Chord diagram illustrating KEGG enrichment terms for down‐regulated and their corresponding genes. H) Gene set enrichment analysis (GSEA) for gene sets in oxidative phosphorylation and citrate cycle (TCA cycle) between PBS and Que@MOF/Man group. NES, nominal enrichment score.

To further investigate the mechanisms underlying the antioxidant and immunoregulatory effects of Que@MOF/Man on macrophages, transcriptomic analysis was performed on LPS‐induced RAW 264.7 cells treated with PBS, Que@MOF/Man and free Que to evaluate mRNA expression differences. Principle component analysis (PCA) revealed distinct mRNA expression patterns for Que@MOF/Man and free Que treatments compared to PBS treatment (**Figure** **7**A; Figure S10, Supporting Information). A total of 1237 differentially expressed genes (DEGs) were identified in cells treated with Que@MOF‐8/Man compared to PBS, with 776 up‐regulated and 461 down‐regulated genes (Figure 7B; Figure S11, Supporting Information). In contrast, cells treated with free Que exhibited 706 up‐regulated genes, and 348 down‐regulated genes compared to PBS‐treated cells (FigureS12, Supporting Information). Notably, only one gene was found to be down‐regulated in cells treated with Que@MOF/Man compared to those treated with free Que (Figure S13, Supporting Information). These results indicated that Que@MOF/Man and free Que have similar potential to induce significant changes in gene expression profiles, suggesting that the carrier and surface modification do not compromise the effective impact of Que on cellular responses.

Subsequently, Gene Ontology (GO) functional annotation and enrichment analyses were performed to elucidate the biological processes associated with DEGs. Among the relevant top 10 enriched terms of down‐regulated genes, there was a notable emphasis on immune‐related processes, particularly those involving responses to oxidative stress, regulation of cytokine production within immune responses, inflammatory responses, and immune responses (Figure 7C). These findings highlighted a significant downregulation of oxidative stress‐ and immune‐related genes in the presence of Que@MOF/Man compared to PBS. This suggested that Que@MOF/Man might exert a substantial influence on the expression patterns of genes in RAW 264.7 cells, leading to a reduction in oxidative stress and suppression of inflammatory responses, which was consistent with in vitro experimental outcomes. Furthermore, the relevant top 10 enriched terms among the up‐regulated genes following Que@MOF/Man treatment included biological processes related to antigen processing and presentation (Figure 7D), which were not observed in the free Que treatment (Figure S14, Supporting Information). This observation suggested that Man modification may enhance endocytosis in RAW 264.7 cells, thereby positively contributing to the modulation of immune responses.

Additionally, the Kyoto Encyclopedia of Genes Genomes (KEGG) pathway enrichment analysis was performed to determine the pathways associated with DEGs. The relevant top enriched KEGG pathways for down‐regulated DEGs following Que@MOF/Man treatment were primarily involved in signaling pathways such as tumor necrosis factor (TNF), interleukin 17 (IL‐17), NF‐κB, janus kinase‐signal transducers and activators of transcription (JAK‐STAT) and mitogen‐activated protein kinase (MAPK) signaling pathways (Figure 7E). These pathways are well‐documented for their roles in immunoregulation, and the secretion of cytokine and chemokine.^[^ ^44^ ^]^ Accordingly, these results indicated that Que@MOF/Man could effectively attenuate LPS‐induced inflammation and promote inflammation resolution via immune regulation. The chord diagram revealed that the down‐regulated genes were predominantly those related to pro‐inflammatory cytokines and chemokines (Figure 7F), particularly *Tnf*, *Il1b*, and *Il6*, corroborating the reduced secretion of pro‐inflammatory cytokines observed in vitro experiments. Moreover, the relevant top‐enriched KEGG pathway for up‐regulated DEGs indicated that Que@MOF/Man might have a positive association with the DEGs involved in cell adhesion and ECM‐receptor interaction (Figure 7G), potentially contributing to the macrophage‐mediated promotion of cardiomyocyte proliferation.^[^ ^45^ ^]^

Specific genes screened from KEGG terms were further analyzed using a protein‐protein interaction network (Figure S15, Supporting Information). Notably, insulin‐like growth factor‐1 (IGF‐1, encoded by *Igf1*), an important regulator of the immune system secreted by anti‐inflammatory M2 macrophages, facilitates a reparative M2 macrophage activation program.^[^ ^46^ ^]^ IGF‐1 is also known to play a crucial role in cardiomyocyte proliferation.^[^ ^47^ ^]^ Intriguingly, gene set enrichment analysis (GSEA) revealed significant activation of oxidative phosphorylation and citrate cycle (TCA cycle) pathways in cells treated with Que@MOF/Man, with enrichment scores of 1.377 and 1.468, respectively (Figure 7H). This suggests that Que@MOF/Man treatment might have a potential influence on the energy metabolic function of macrophages, which plays an important part in macrophage polarization indicated by previous study.^[^ ^48^ ^]^ Taken together, these results highlight the pivotal role of Que@MOF/Man in attenuating oxidative stress, promoting macrophage differentiation toward a regenerative phenotype, and enhancing inflammation resolution, thereby stimulating tissue repair potential.

### 2.8 In Vivo Targeted Delivery to Inflamed Myocardium and Anti‐Inflammatory Effects

**Figure 8:**
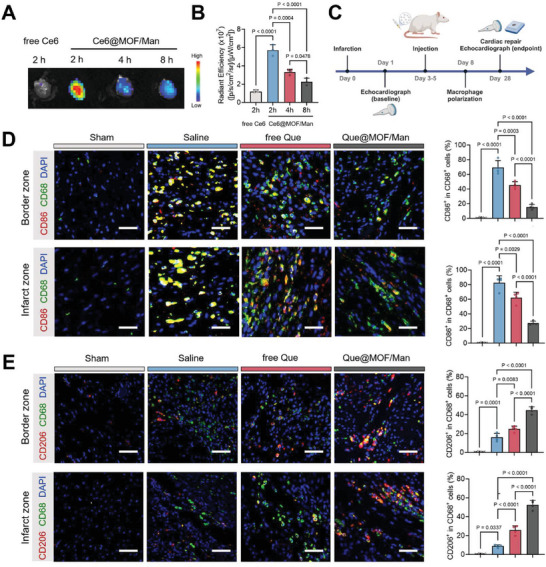
In vivo biodistribution and anti‐inflammatory effects of Que@MOF/Man. A) Schematic timeline of the in vivo experimental protocol. B,C) Representative ex vivo fluorescence images (B) and quantification analysis (C) for free Ce6 and Ce6@MOF/Man accumulation in the infarcted hearts of MI rats at different timepoints (2, 4, and 8 h) after intravenous injection (*n* = 3). D,E) Representative confocal images (D) and quantification analysis (E) for the proportion of M1 phenotype (CD86^+^) and M2 phenotype (CD206^+^) macrophages within the total macrophages population (CD68^+^) in infarcted heart sections of MI rats after different treatments, with sham surgery serving as the control. (*n* = 4). Scalar bar, 50 µm. Data are presented as mean ± SD.

To determine the inflammation‐targeting efficiency, the biodistribution of free Ce6 and Ce6‐labeled nanoparticles in the inflamed hearts of rats was first investigated at different time points (2, 4, and 8 h) following tail vein injection. Ex vivo fluorescence imaging revealed a notable fluorescent accumulation in infarcted hearts of the Ce6@MOF/Man group, with a 4.8‐fold higher MFI at 2 h post‐injection compared to the free Ce6 group (**Figure** **8**A,B). This result was further confirmed by myocardium slices (Figure S16, Supporting Information). Notably, strong signals were exclusively detected in the livers of free Ce6‐treated MI rats, indicating rapid clearance from systemic circulation. However, a preferential accumulation in the hearts of Ce6@MOF/Man‐treated MI rats was observed (Figure S17, Supporting Information). We further performed a quantitative analysis for the targeting capability of Ce6@MOF/Man, employing the heart‐targeting index (HTI), defined as the ratio of heart radiant efficiency to liver radiant efficiency.^[^ ^49^ ^]^ The HTI value of free Ce6 at 2 h post‐injection (0.35) was significantly lower than those of all the detected samples in Ce6@MOF/Man groups: 1.28 for 2 h, 1.38 for 4 h and 0.69 for 8 h post‐injection (Figure S17, Supporting Information). These observations suggested that Ce6@MOF/Man achieves the enhanced heart‐targeting ability to inflame the myocardium, likely due to the inflammation‐based EPR effect induced by MI. Intriguingly, the ex vivo fluorescence intensity progressively diminished from 2 to 8 h post‐injection (Figure 8A,B), indicating that the nanoparticles exhibit an optimal circulation time to exert their therapeutic effects.

Following the confirmation of its targeting ability to the inflamed myocardium, the inflammatory modulation performance of Que@MOF/Man was evaluated. MI rats were randomly assigned to receive intravenous injections of saline, free Que or Que@MOF/Man at 3 days post‐MI, with injections administered 3 times in succession. Rats that underwent thoracotomy without MI injury served as the sham control. At 3 days post‐treatment (8 days post‐MI), the rats were euthanized, and heart samples from different groups were collected for immunofluorescence staining to investigate macrophage polarization mediated by Que@MOF/Man (Figure 8C). The confocal images showed a high expression of CD86 (a specific marker for M1 macrophages) among CD68^+^ macrophages (representing the total macrophage population) in both the infarct and border zones at 3 days post‐treatment in the saline group, with a corresponding low expression of CD206 (a specific marker for M2 macrophages) (Figure 8D,E). Notably, Que@MOF/Man treatment significantly altered the macrophage subtype distribution, with an observed increase in the CD206^+^ population and a decrease in the CD86^+^ population in both the infarct and border zones of the injured hearts. These results collectively indicated that the targeted delivery of Que@MOF/Man to injured hearts in MI rats has the potential to shift the inflammatory response toward an anti‐inflammatory state, potentially inhibiting the infiltration of inflammatory cells and promoting cardiac repair.

### 2.9 Cardiac Repair Against MI

**Figure 9:**
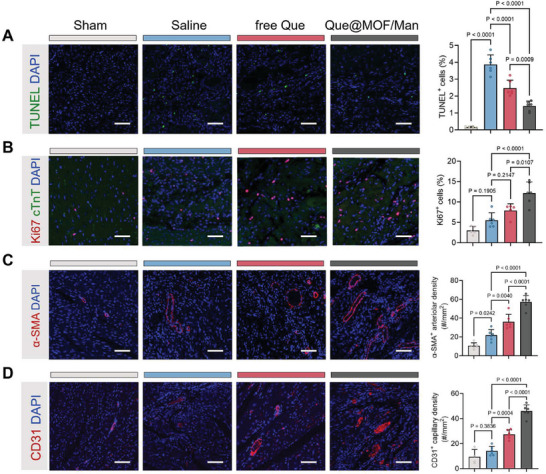
Que@MOF/Man‐mediated in vivo inflammation resolution on cardiomyocyte survival and angiogenesis. A,B) Representative confocal images and quantification analysis for TUNEL^+^ (A) and Ki67^+^ (B) cardiomyocytes in the border zone of infarcted hearts following different treatments (*n* = 6). Scale bar, 50 µm. C,D) Representative confocal images and quantification analysis for α‐SMA^+^ (C) and CD31^+^ (D) vessels in the border zone of infarcted hearts after different treatments (*n* = 4–6). Scale bar, 50 µm. Data are presented as mean ± SD.

Cardiac repair is critically dependent on cardiomyocyte survival. To this end, we initially examined the impact of inflammation resolution on cardiomyocyte apoptosis and proliferation in the border regions of infarcted hearts at 28 days post‐MI. TUNEL staining revealed that MI injury resulted in a 3.87% increase in cardiomyocyte apoptosis compared to the sham group (**Figure** **9**A). Following treatment with free Que and Que@MOF/Man, the number of TUNEL^+^ cardiomyocytes was remarkably decreased to 2.47% and 1.42%, respectively. Moreover, cTnT^+^ cardiomyocytes displaying Ki67 signals in nuclei labeled with DAPI were identified, indicating active cell division and proliferation. Notably, the proportion of Ki67^+^ cardiomyocytes was more pronounced in the border zone of Que@MOF/Man‐treated hearts than in other groups, suggesting that this treatment enhanced cardiomyocyte cycling and significantly rescued cardiomyocyte viability (Figure 9B). These findings can be potentially attributed to cellular interaction and communication, such as the effects of anti‐inflammatory cytokines and other molecules secreted by Que@MOF/Man‐induced reparative macrophages on cardiomyocytes. Driven by the cardioprotective effects of Que@MOF/Man on inflammation regulation, the extent of angiogenesis was also assessed. Immunofluorescence staining with alpha‐smooth muscle actin (α‐SMA) and CD31 was conducted at 28 days post‐MI to evaluate the arteriolar and capillary density, respectively. The representative fluorescent images and quantitative analysis revealed that the α‐SMA^+^ arteriolar density and CD31^+^ capillary density in the border zone of the Que@MOF/Man group were 2.62‐fold and 3.26‐fold higher than those in the saline group, respectively (Figure 9C,D). These results indicated that the regenerative inflammation microenvironment fostered angiogenesis and blood vessel preservation post‐MI, which was consistent with the previous studies.^[^ ^50^ ^]^

**Figure 10:**
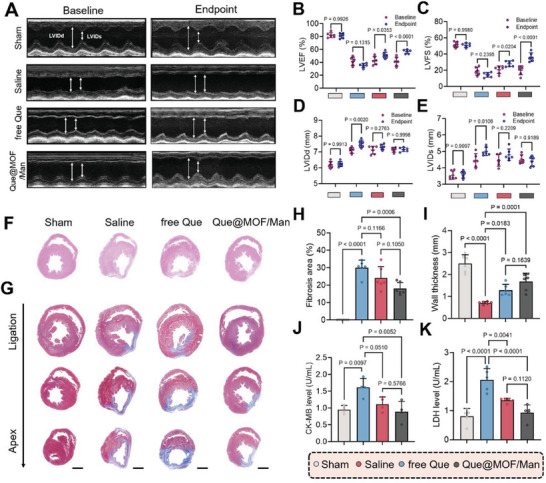
Cardiac repair efficacy of Que@MOF/Man in MI rats. A) Representative M‐mode echocardiographic images at 1 day post‐MI (baseline) and 28 days post‐MI, with arrows indicating left ventricular dimension at diastole (LVIDd) and systole (LVIDs). B–E) Quantification analysis for left ventricular ejection fraction (LVEF, B), left ventricular fractional shortening (LVFS, C), LVIDd (D), and LVIDs (E) after different treatments at baseline and 28 days post‐MI. (*n* = 6). F) Representative bright field images of inflammation infiltration in heart sections, as assessed by H&E staining, following various treatments. Scale bar, 2 mm. G–I) Representative bright field images of infarcted hearts (G), and quantification analysis for fibrosis area (H) and wall thickness (I) via Masson's trichrome staining after different treatments at 28 days post‐MI (*n* = 6). Blue and red areas denote fibrosis and myocardium, respectively. Scale bars, 2 mm. J,K) Serum levels of CK‐MB (*n* = 4) and LDH (*n* = 5) after different treatments at 28 days post‐MI. Data are presented as mean ± SD.

Considering the strengthened reparative capacity post‐MI associated with an increased presence of anti‐inflammatory macrophages,^[^ ^51^ ^]^ we further investigated the cardiac function using echocardiography at 1 day (baseline) and 28 days post‐MI. Quantitative analysis indicated a notable reduction in left ventricular ejection fraction (LVEF) from 41.5% to 35.1% and left ventricular fractional shortening (LVFS) from 18.95% to 14.25% in the saline group. However, the administration of free Que and Que@MOF/Man mitigated the further deterioration of cardiac ejection function, with the Que@MOF/Man group showing a significant improvement (*p* < 0.0001) compared to the saline group (**Figure** **10**B,C). Moreover, we measured the cardiac dimensions, including the left ventricular internal diameter at end‐diastole (LVIDd) and the left ventricular internal diameter at end‐systole (LVIDs). A notable increase in both LVIDd (from 7.11 to 7.52 mm) and LVIDs (from 4.44 to 5.01 mm) was observed in MI rats of the saline group (Figure 10D,E), indicating a heightened risk of myocardial dilatation post‐MI. The cardiac dimensions remained relatively stable following treatment with free Que and Que@MOF/Man, with the latter further preventing myocardial remodeling. Consistent with the worsening cardiac function, the histological evaluation by the hematoxylin and eosin (H&E) staining and the Masson's trichrome staining of heart sections revealed a larger fibrosis area and less retained myocardium in the saline group (Figure 10F,G). In contrast, the Que@MOF/Man group exhibited a fibrosis area approximately 1.67‐fold smaller than that of the saline group, and a significantly thicker myocardium (*p* = 0.0001, Figure 10H,I). Furthermore, we evaluated the creatine kinase‐MB (CK‐MB) and serum lactate dehydrogenase (LDH), commonly used indicators of myocardial damage in clinical practice. Despite a non‐significant reduction in CK‐MB (*p* = 0.2080) and LDH (*p* = 0.1456) levels in the Que@MOF/Man group at 3 days post‐treatment (8 days post‐MI) (Figure S18, Supporting Information), a pronounced inhibitory effect on both CK‐MB and LDH levels was observed in MI rats treated with Que@MOF/Man at 28 days post‐MI (Figure 10J,K).

Moreover, the potential toxicity of the treatments was investigated. Serum biochemistry analysis for hepatic and renal function demonstrated no significant differences among all groups (Figure S19, Supporting Information), and no histopathological changes were observed in major organs (Figure S20, Supporting Information) at 28 days post‐MI. In summary, these results suggested that Que@MOF/Man could be used as a safe inflammation‐targeting delivery approach that effectively enhances cardiomyocyte survival and angiogenesis, thereby improving cardiac function post‐MI.

## 3 Conclusion

In summary, we have successfully developed Que@MOF/Man nanoparticles for targeted delivery to the inflamed myocardium and macrophages, aiming to mitigate oxidative stress and regulate inflammation response for cardiac repair. Leveraging the dual anti‐oxidative and anti‐inflammatory properties of quercetin, Que@MOF/Man effectively alleviated oxidative stress and resolved inflammation in macrophages. This interaction further protected cardiomyocytes from oxidative stress, which in turn protected cardiomyocytes from further oxidative damage. Following systemic administration, Que@MOF/Man exhibited enhanced accumulation at the site of myocardial injury, effectively curbing the progression of inflammation and facilitating its resolution. This led to a significant improvement in cardiomyocyte survival and vascular preservation. Ultimately, this treatment strategy exhibited remarkable efficacy in preventing adverse myocardial remodeling and improving cardiac function. Overall, our research presents a promising therapeutic alternative for myocardial infarction, characterized by favorable biocompatibility and outstanding therapeutic outcomes.

## 4 Experimental Section

### Materials

Mannan and Sodium nitroferricyanide (III) dihydrate (SNP) were purchased from Sigma‐Aldrich (MO, USA). 2‐methylimidazole, Zn(NO_3_)_2_·6H_2_O, quercetin,2,2‐Diphenyl‐1‐picrylhydrazyl (DPPH) and 2,2′‐Azino‐bis(3‐ethylbenzthiazoline‐6‐sulfonic acid) (ABTS) were purchased from Aladdin (Shanghai, China). Dulbecco's Modified Eagle's Medium (DMEM), fetal bovine serum (FBS), penicillin−streptomycin, and trypsin were purchased from Gibco (NY, USA). Inhibition and produce superoxide anion assay kit, hydroxyl free radical assay kit, and MDA assay kit were obtained from Nanjing Jiancheng Bioengineering Institute (Nanjing, China). The enzyme‐linked immunosorbent assay (ELISA) kits of mouse TNF‐α, IL‐1β, IL‐6, and IL‐10 were purchased from R&D systems (MN, USA).

### Synthesis of Que@MOF/Man

2‐methylimidazole (2.5 g, 61 mmol) was fully dissolved in 25 mL methanol solution. Zn(NO_3_)_2_·6H_2_O (0.25 g, 0.83 mmol) were dissolved in 5 mL methanol solution and added into the above 2‐methylimidazole solution dropwise and stirred for 15 min at room temperature. The products were washed with methanol 3 times at 12000 rpm for 15 min. The ZIF‐8 nanoparticles were obtained and stored in methanol. The ZIF‐8 methanol solution was mixed with 5 mL of Que (15 mg, 0.05 mmol) methanol solution. Then the mixture was stirred for 15 min and the Que@MOF nanoparticles were collected by centrifugation. Successively, Que@MOF methanol solution was added into Man aqueous solution (5 mg mL^−1^, 20 mL) and further stirred for 60 min. Then, Que@MOF/Man was collected by centrifugation and washed 3 times by a methanol aqueous solution. Finally, the Que@MOF/Man nanoparticles were obtained by vacuum drying.

### Characterization of Que@MOF/Man

The as‐prepared ZIF‐8, Que@MOF, and Que@MOF/Man nanoparticles were characterized by different microscopic and spectroscopic analyses. Briefly, the size distribution and zeta potential of nanoparticles were measured by dynamic light scattering (DLS) analysis (Zetasizer Nano ZSP, Malvern, UK). The morphology was detected by TEM (Tecnai G2 F20 S‐TWIN, FEI, USA). To examine the crystalline structure, X‐ray diffraction (XRD) analysis was conducted (Rigaku, Tokyo, Japan). The chemical structure and composition were characterized by Fourier‐transform infrared spectroscopy (FTIR) (Nicolet iS50, Thermo Scientific, USA) and X‐ray photoelectron spectroscopy (XPS) (XSAM800, Kratos Analytical, UK), respectively. The Que content in Que@MOF/Man (first dispersed in pH 1 HCl and diluted with methanol) was determined by measuring the absorbance at 374 nm and referring to a standard curve of free quercetin in methanol obtained by the UV–Vis spectrophotometer (UV‐2600, SHIMADZU, Japan).

### DPPH Free Radical Scavenging Activity

Different concentrations of Que@MOF/Man (50 µL) and PBS buffer (150 µL, pH 5.5 and pH 7.4) were added into DPPH ethanol solution (0.3 mM, 200 µL). The mixed solution was vortexed and incubated in the dark. The UV–Vis absorbance was monitored every 2 for 30 min at 517 nm and UV–Vis spectra of 400–700 nm were measured at 30 min. The standard curve was prepared to qualify DPPH using UV–Vis absorbance at 517 nm, based on which DPPH scavenging capacity was estimated.

### ABTS^•+^ Free Radical Scavenging Activity

To prepare the ABTS^•+^ solution, 7.4 mM ABTS, and 2.6 mM K_2_S_2_O_8_ were mixed with equal volume in the PBS (pH 7.4) and then stored in the dark at 4 °C overnight. The ABTS^•+^ working solution was obtained by diluting the concentrated solution with PBS (pH 7.4) to an absorbance of 0.70 ± 0.02 at 734 nm. Then, different concentrations of quercetin, Que@MOF or Que@MOF/Man (20 µL), and PBS buffer (80 µL, pH 5.5 and pH 7.4) were added into the ABTS^•+^ working solution (400 µL). The UV–Vis absorbance was monitored every 2 min for 30 min at 734 nm and UV–Vis spectra of 500–900 nm were measured at 30 min. Similarly, the ABTS^•+^ scavenging capacity was calculated based on a standard curve for ABTS^+•^.

### Hydroxyl, Superoxide, and Nitric Oxide Radicals scavenging Activity

The hydroxyl radical (•OH) and superoxide Radical (•O_2_^−^) scavenging activity of Que@MOF/Man was measured by using the hydroxyl free radical assay kit and inhibition and produce superoxide anion assay kit, respectively, according to the manufacturer's instructions. The nitric oxide radical (•NO) was produced by sodium nitroprusside (SNP) and measured by nitric oxide assay kit. Briefly, SNP (0.5 M, 100 µL) and different concentrations of Que@MOF/Man (100 µL) were mixed in 200 µL PBS, followed by shaking in 37 °C for 90 min. Then, 50 µL of the sample, Griess reagent R1 and R2 were added for 10 min, and the UV–Vis spectra of 400–700 nm were measured.

### Cell Culture

Mouse monocytes/macrophages cell line (RAW 264.7 cells) and rat cardiomyocytes cell line (H9C2 cells) were originally obtained from American Type Culture Collection (ATCC) and cultured in high‐glucose DMEM containing 10% FBS and 1% penicillin‐streptomycin at 37 °C with 5% CO_2_.

### Cell Viability Assay

CCK‐8 assay was used to detect the cytotoxicity of Que@MOF/Man to RAW 264.7 cells. Briefly, RAW 264.7 cells (1 × 10^4^ per well) were seeded in 96‐well plates. After cell adherence, various concentrations of Que@MOF/Man were successively added to each well and incubated for 4 h, which were then replaced with fresh culture media for another 20 h. The relative cell viability was determined by CCK‐8 assay.

### Hemolysis Test

Blood samples from rats were extracted by aorta ventralis puncture, centrifuged erythrocytes were aspirated, and saline was added in the appropriate ratio (1:49) to make a final concentration of 2% of the erythrocyte suspension. The erythrocyte suspension was then combined with ZIF‐8 and Que@MOF/Man, which were diluted to gradient concentrations of 50, 100, 150, and 200 µg mL^−1^, and incubated at 37 °C for 1 h. The supernatants of samples were centrifuged, and the rate of hemolysis was measured. With ddH_2_O serving as a positive control and saline as a negative control, the hemolysis rate was represented as a percentage of the ddH_2_O group.

### Cellular Uptake

To evaluate the selective uptake of Que@MOF/Man by macrophages and cardiomyocytes, the RAW 264.7 cells (1 × 10^4^ cells/well) and H9C2 cells (5 × 10^3^ cells/well) were employed in a 96‐well plate. Furthermore, different activations were used to induce RAW 264.7 cells to mimic macrophages in different states under inflammatory microenvironments. M0 macrophages were achieved by treating RAW 264.7 cells with PBS. M1 macrophages were achieved by treating RAW 264.7 cells with LPS (300 ng mL^−1^) for 6 h. M2 macrophages were achieved by treating RAW 264.7 cells with IL‐4 (20 ng mL^−1^) and IL‐13 (20 ng mL^−1^) for 24 h. As a fluorescence probe, Ce6 was packaged instead of Que in Ce6@MOF/Man to track the uptake process. Then, cells were treated with free Ce6 and Ce6@MOF/Man in serum‐free cell culture medium (Ce6 concentration: 1.25 µg mL^−1^, excitation: 633 nm). After 1, 2, or 4 h of incubation at 37 °C, the cells were washed thrice with ice‐cold PBS and fixed with immune staining fix solution for 15 min. Successively, the nuclei were stained with Hoechst 33342 (1 µg mL^−1^, excitation: 405 nm) for 10 min and washed thrice with ice‐cold PBS. Then, the cells were photographed using the Opera Phenix Plus confocal microscope (PerkinElmer, USA).

Further, to quantify the cellular uptake level, the cells were seeded in 12‐well plate (1 × 10^5^ cells/well), and the same treatments were performed as described above. The cells were collected after being washed with ice‐cold PBS thrice and then dispersed in ice‐cold PBS for flow cytometry analysis (NovoCyte, ACEA Biosciences, USA).

### Intracellular ROS Scavenging

The intracellular levels of total ROS were assessed using DCFH‐DA as the indicator. RAW 264.7 cells were seeded in a 96‐well plate (1 × 10^4^ cells/well) and treated with LPS (300 ng mL‐1) for 6 h to mimic the inflammatory state. Non‐inflamed cells served as the control. Then, LPS‐induced cells were treated with PBS, free Que, and Que@MOF/Man (Que concentration: 1.25 µg mL^−1^) in serum‐free cell culture medium for 4 h. The drug‐containing medium was refreshed with 10% FBS‐containing medium for another 20 h. Then, DCFH‐DA (10 µM) was added and incubated with cells for 30 min and Hoechst (1 µg mL‐1) was added to stain the cell nuclei for 10 min followed by fluorescence microscope observation.

Moreover, flow cytometry was also used to collect quantitative data about the intracellular ROS level. The cells were seeded in a 12‐well plate (1 × 10^5^ cells/well), and the same treatments were performed as fluorescence imaging. Then cells were harvested, and the fluorescence intensity was measured by flow cytometry (NovoCyte, ACEA Biosciences, USA).

### Intracellular Lipid Peroxidation Activity

The lipid peroxidation activity was measured by MDA (an important product of membrane lipid peroxidation) assay. RAW 264.7 cells were seeded in a 12‐well plate (1 × 10^5^ cells/well) and treated with LPS (300 ng mL^−1^) for 6 h to mimic the inflammatory state. Non‐inflamed cells served as the control. Then, LPS‐induced cells were treated with PBS, free Que, and Que@MOF/Man (Que concentration: 1.25 µg mL^−1^) in serum‐free cell culture medium for 4 h. The drug‐containing medium was refreshed with 10% FBS‐containing medium for another 20 h. Subsequently, RAW 264.7 cells were collected and followed by adding the extraction reagent. The supernatants were collected after centrifugation at 12 000 g for 10 min, and the protein concentration was quantified. The supernatants mixed with MDA detection reagents were kept at 100 °C for 1 h. After cooling down and centrifugation, the absorbance values at 532 nm were calculated.

### Intracellular Superoxide Dismutase (SOD) Activity

The SOD was measured by WST‐8 assay. Briefly, RAW 264.7 cells were seeded in a 12‐well plate (1 × 10^5^ cells/well) and treated with LPS (300 ng mL^−1^) for 6 h to mimic the inflammatory state. Non‐inflamed cells served as the control. Then, LPS‐induced cells were treated with PBS, free Que, and Que@MOF/Man (Que concentration: 1.25 µg mL^−1^) in serum‐free cell culture medium for 4 h. The drug‐containing medium was refreshed with 10% FBS‐containing medium for another 20 h. Then the RAW 264.7 cells were collected and fragmented after adding the extraction reagent. The supernatants were collected by centrifugation, and the protein concentration was quantified. The assay reagent was then added and incubated for 30 min at 37 °C. At 450 nm, the absorbance values were calculated.

### In Vitro Macrophage Polarization

To verify the effects of Que@MOF/Man nanoparticles on the polarization state of macrophages in vitro, flow cytometry, real‐time, western blot analysis, immunofluorescence staining, and the ELISA assay were performed.

The flow cytometry was used to detect the expression of the surface marker for macrophage polarization. RAW 264.7 cells were seeded in a 12‐well plate (1 × 10^5^ cells/well) and activated by LPS (300 ng mL^−1^) for 6 h. Non‐inflamed macrophages served as the control. LPS‐induced cells were treated with PBS, free Que and Que@MOF/Man (Que concentration: 1.25 µg mL^−1^) in serum‐free cell culture medium for 4 h. Subsequently, the cell culture medium was refreshed with 10% FBS‐containing medium for another 20 h. The macrophages were collected and incubated with anti‐mouse CD16/32 on ice for 10 min followed by incubation of a 1% antibody mixture (FITC anti‐mouse CD11b and APC anti‐mouse CD86) on ice for 30 min. After the fixation and permeabilization, cells were successively stained with PE‐Cy7 anti‐mouse CD206 at room temperature for 30 min. Then cells were resuspended with 300 µL PBS. Flow cytometry analysis was performed using a Beckman Coulter platform (Life Science, USA).

The polarization state of macrophages in vitro was further confirmed by immunofluorescence assay. RAW 264.7 cells were seeded in a 96‐well plate (1 × 10^4^ cells/well), and the same treatments were performed as above. Then cells were fixed in 4% paraformaldehyde and permeabilized with 0.1% Briji. Next, cells were blocked using 20% goat serum and further incubated with anti‐iNOS (1:100, GeneTex) and anti‐Arg‐1 (1:100, GeneTex) at 4 °C overnight, followed by TRITC‐conjugated goat anti‐rabbit secondary antibody (1:200, Jackson). The cytoskeleton and nuclei were stained with Alexa Fluor 488‐conjugated phalloidin and DAPI, respectively. The immunofluorescence sections were imaged by the Opera Phenix Plus confocal microscope (PerkinElmer, USA).

The western blot analysis was used to detect the relative expression of protein related to macrophage polarization. RAW 264.7 cells were seeded in a 12‐well plate (1 × 10^5^ cells/well) and the same treatments were performed as described above. The cells were lysed with RIPA buffer containing protease and phosphatase inhibitor cocktails, and the protein content was quantified by using a BCA kit (Thermo, USA). The protein samples were subjected to sodium dodecyl sulfate‐polyacrylamide gel electrophoresis (SDS‐PAGE) and transferred to polyvinylidene fluoride (PVDF) membranes (Millipore, USA). Then, the PVDF membranes were blocked with 5% nonfat milk for 1 h at room temperature and washed 3 times using Tris‐buffered saline plus Tween (TBST). Next, the PVDF membranes were incubated at 4 °C overnight with specific primary antibodies. Primary antibodies were diluted as follows: iNOS (1:1000, GeneTex), Arg‐1 (1:1000, GeneTex), and β‐Tubulin (1:5000, proteintech). The next day, the PVDF membranes were washed with TBST and then incubated with anti‐rabbit IgG horseradish‐peroxidase‐conjugated secondary antibodies at room temperature for 1 h. Immunoreactive bands were visualized using an immobilized western chemiluminescent HRP substrate (Millipore, USA) and a chemiluminescence imager (Bio‐Rad, USA). Relative protein expression levels were normalized to β‐Tubulin. Images were quantified using ImageJ software.

The ELISA was used to detect the secretion of pro‐inflammation and anti‐inflammation cytokines. RAW 264.7 cells were seeded in a 12‐well plate (1 × 10^5^ cells/well) and the same treatments were performed as described above. Then, the supernatants were collected and the concentrations of pro‐inflammation cytokines (IL‐1β, IL‐6, and TNF‐α) and anti‐inflammation cytokines (IL‐10) were measured with corresponding ELISA kits (R&D systems, USA).

The real time‐qPCR was used to detect the relative mRNA expression levels of M1 macrophage markers (iNOS, IL‐6, IL‐1β, and TNF‐α) and M2 macrophage markers (TGF‐β, IL‐10, Arg‐1, CD206, and CD163). The total RNA of macrophages was extracted by TRIzol (Takara, Japan) according to the manufacturer's instruction, and reverse transcription of mRNA was performed utilizing the PrimeScript RT Reagent Kit (Takara, Japan). Through the application of SYBR GREEN reagent (Thermo, USA), the gene expression factors relevant to the M1 phenotype and M2 phenotype were detected by the QuantStudio3 PCR system (Thermo, USA). Gene expression levels in all samples were normalized using the 2^−ΔΔCt^ method with GAPDH as internal controls for comparison.

### Cardioprotective Effects of Que@MOF/Man‐Treated Macrophages

To examine the protective effects of macrophages under different treatments on cardiomyocytes, the intracellular ROS level, lipid peroxidation activity, SOD activity, and cell viability of injured cardiomyocytes were inspected.

The intracellular levels of total ROS in injured cardiomyocytes were assessed using DCFH‐DA as the indicator. H9C2 cells were seeded on a 24‐well plate (5 × 10^4^ cells/well) in a normal or ROS microenvironment induced by 400 µM H_2_O_2_ for 24 h. RAW 264.7 cells were seeded on transwells and treated with LPS (300 ng mL^−1^) for 6 h. Then, LPS‐induced cells were treated with PBS, free Que and Que@MOF/Man (Que concentration: 1.25 µg mL^−1^) in serum‐free cell culture medium for 4 h. Subsequently, the cell culture medium was refreshed with 10% FBS‐containing medium for another 20 h. The transwells were put into the 24‐well plate seeded with H_2_O_2_‐treated H9C2 cells (Figure 6A). After 48‐h co‐incubation, the transwells were removed. Then, DCFH‐DA (10 µM) was added in H9C2 cells on a 24‐well plate and incubated with cells for 30 min and Hoechst (1 µg mL^−1^) was added to stain the cell nuclei for 10 min followed by fluorescence microscope observation.

To confirm the lipid peroxidation activity in injured H9C2 cells, the MDA assay was performed, and the same treatments were performed as above. Subsequently, H9C2 cells were collected and followed by adding the extraction reagent. The supernatants were collected after centrifugation at 12 000 g for 10 min, and the protein concentration was quantified. The supernatants mixed with MDA detection reagents were kept at 100 °C for 1 h. After cooling down and centrifugation, the absorbance values at 532 nm were calculated.

The SOD activity of H9C2 cells was also assessed. Normal and H_2_O_2_‐injured H9C2 cells were similarly co‐cultured with RAW 264.7 cells in different states for 48 h. Then the H9C2 cells were collected and fragmented after adding the extraction reagent. The supernatants were collected by centrifugation, and the protein concentration was quantified. The assay reagent was then added and incubated for 30 min at 37 °C. At 450 nm, the absorbance values were calculated.

### Myocardial Infarction Injury Model in Rats

Male SD rats (250 ± 20 g, 8–10 weeks old) were purchased from Beijing HFK Bioscience Co., Ltd (Beijing, China) and maintained in a specific pathogen‐free (SPF)‐class animal room. All animals were handled in accordance with protocols approved by the Sichuan University Laboratory Animal Center and with an internal ethics board (20220512377). Rats were continuously anesthetized with 2% isoflurane and intubated with an 18‐gauge intravenous catheter. Under aseptic conditions, the heart was exposed by left open thoracotomy, myocardial infarction was achieved by the permanent ligation of the left anterior descending artery (LAD) with 7‐0 polyester suture. The injury of the hearts was confirmed by ST‐segment characterized electrocardiogram and left ventricle color alteration. Then the MI rats were treated with saline, free Que, and Que@MOF/Man (dose: 1 mg kg^−1^) through tail vein injection. The injections were performed at 3 days post‐MI of 3 successive times. The sham rats served as the surgical control and were subjected to the same procedure as MI rats without LAD ligation.

### Ex Vivo Biodistribution and Targeting Specificity

For imaging the biodistribution of the particles, Ce6‐loaded particles were first prepared by replacing Que to visually track the cellular uptake process. After MI surgery for 3 days, free Ce6 and Ce6@MOF/Man were intravenously injected into MI rats. After 2, 4, and 8 h post‐injection, the hearts and major organs (livers, spleens, lungs, and kidneys) were harvested from the rats, and subsequently, ex vivo imaging was acquired using an IVIS Lumina III imaging system. Then, hearts were cut into 1‐mm‐thick slices, and ex vivo imaging was performed on the slices. The mean signal intensity was quantified by measuring ROI using the Living Image 3.1 software.

### Therapeutic Efficacy in MI Rats

MI‐induced SD rats were blindly divided into 3 groups and the groups were injected saline, free Que, and Que@MOF/Man respectively, through the tail vein at 3 days post‐MI. The sham rats served as the control. Blood samples in different groups were collected at different time‐points (8 days post‐MI and 28 days post‐MI), and the plasma lactate dehydrogenase (LDH) and creatine kinase isoenzyme (CK‐MB) levels were measured. Cardiac geometry structure and function were assessed using the 2D guided M‐mode echocardiography (Vevo 3100, FUJIFILM Visual Sonics, Canada) at 1 day and 28 days post‐MI. The left ventricular ejection fraction (LVEF), left ventricular fractional shortening (LVFS), and left ventricular internal diameter at end‐diastole (LVIDd) and at end‐systole (LVIDs) were obtained in a double‐blind way.

### Histological Analysis

At 28 days post‐MI, the rat hearts in were harvested, fixed with trans‐cardiac perfusion of saline, and immersed in 4% paraformaldehyde overnight to make paraffin sections in several different layers. Masson's trichrome staining was performed to determine the fibrosis area and the ventricular wall thickness. The images were acquired with SLIDEVIEW VS200 microscope (Olympus, Germany) and analyzed using ImageJ software. 3 selected sections were quantified for each animal.

### Immunofluorescence Analysis

To explore the anti‐inflammation effect after treatments, hearts were collected at 3 days post‐treatment. The heart sections were deparaffinized, permeabilized, blocked, and incubated with anti‐CD68, anti‐CD206, and anti‐CD86 to detect M1 and M2 macrophages. Then, sections were incubated with the secondary antibodies and DAPI. The number of CD86^+^, and CD206^+^ macrophages was counted in 3 random microscopic fields per animal.

To evaluate the apoptosis, proliferation, and angiogenesis regulation by Que@MOF/Man, the immunofluorescence staining against TdT‐mediated dUTP nick end labeling (TUNEL), Ki67, alpha‐smooth muscle actin (α‐SMA) and CD31 were conducted at 28 days post‐MI, which was similar to the procedure described above. The number of blood vessels and, apoptotic or proliferative cardiomyocytes were counted using ImageJ software.

### In Vivo Safety Evaluation

At 28 days post‐MI, Hematoxylin, and Eosin (H&E) staining was performed to evaluate histopathological changes in major organs (liver, spleen, lung, and kidney). Blood biochemical indicators were detected using Cobas C 311 automated chemistry analyzer (Roche Diagnostics, Germany), including alanine aminotransferase (ALT) and aspartate aminotransferase (AST), and urea nitrogen (UREA) and creatinine (CREA) indicators.

### Statistical Analysis

All quantitative data were expressed as the means ± standard deviation (SD). The two‐tailed t‐tests and one‐way analysis of variance (ANOVA) followed by Tukey's test were used for comparison. A difference with a *p* value <0.05 was considered statistically significant. And all statistical analyses were performed using GraphPad Prism 8.0.

## Conflict of Interest

The authors declare no conflict of interest.

## Supporting information

Supporting Information

## Data Availability

The data that support the findings of this study are available from the corresponding author upon reasonable request.
