# Supplementary material for: Inflammation‐Targeted Nanomedicines Alleviate Oxidative Stress and Reprogram Macrophages Polarization for Myocardial Infarction Treatment
Source: Adv Sci (Weinh). 2024 Apr 6;11(21):2308910. doi: 10.1002/advs.202308910 (PMC11151042; doi:10.1002/advs.202308910)
Supplement: Supplementary file 1 — Supporting Information [file ADVS-11-2308910-s001.pdf]

ADVANCED SCIENCE
Open Access

Supporting Information

for *Adv. Sci.*, DOI 10.1002/advs.202308910

Inflammation-Targeted Nanomedicines Alleviate Oxidative Stress and Reprogram Macrophages Polarization for Myocardial Infarction Treatment

Danrong Hu, Ran Li, Yicong Li, Meng Wang, Lu Wang, Shiqi Wang, Hongxin Cheng, Qing Zhang, Chenying Fu, Zhiyong Qian and Quan Wei**

SUPPORTING INFORMATION

Inflammation-Targeted Nanomedicines Alleviate Oxidative Stress and Reprogram Macrophages Polarization for Myocardial Infarction Treatment

Danrong Hu[a1], Ran Li[a1], Yicong Li[a], Meng Wang[a], Lu Wang[a], Shiqi Wang[a], Hongxin Cheng[a], Qing Zhang[a], Chenying Fu[b], Zhiyong Qian[a*], Quan Wei[a*]

[a] Rehabilitation Medicine Center and Institute of Rehabilitation Medicine, Key Laboratory of Rehabilitation Medicine in Sichuan Province, State Key Laboratory of Biotherapy and Cancer Center, West China Hospital, Collaborative Innovation Center, Sichuan University, Chengdu, Sichuan, 610041, P. R. China

[b] National Clinical Research Center for Geriatrics, Aging and Geriatric Mechanism Laboratory, West China Hospital, Sichuan University, Chengdu, Sichuan, 610041, P. R. China

[1] These authors contributed equally.

[*] Email: zhiyongqian@scu.edu.cn (Qian ZY); weiquan@scu.edu.cn (Wei Q)

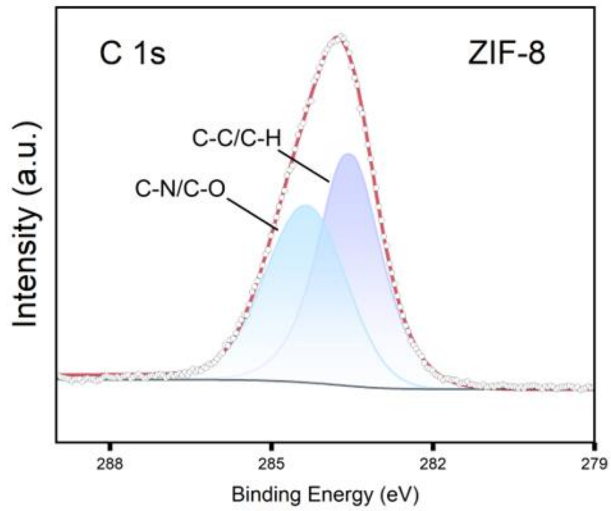

Figure S1. XPS C 1s spectra of ZIF-8.

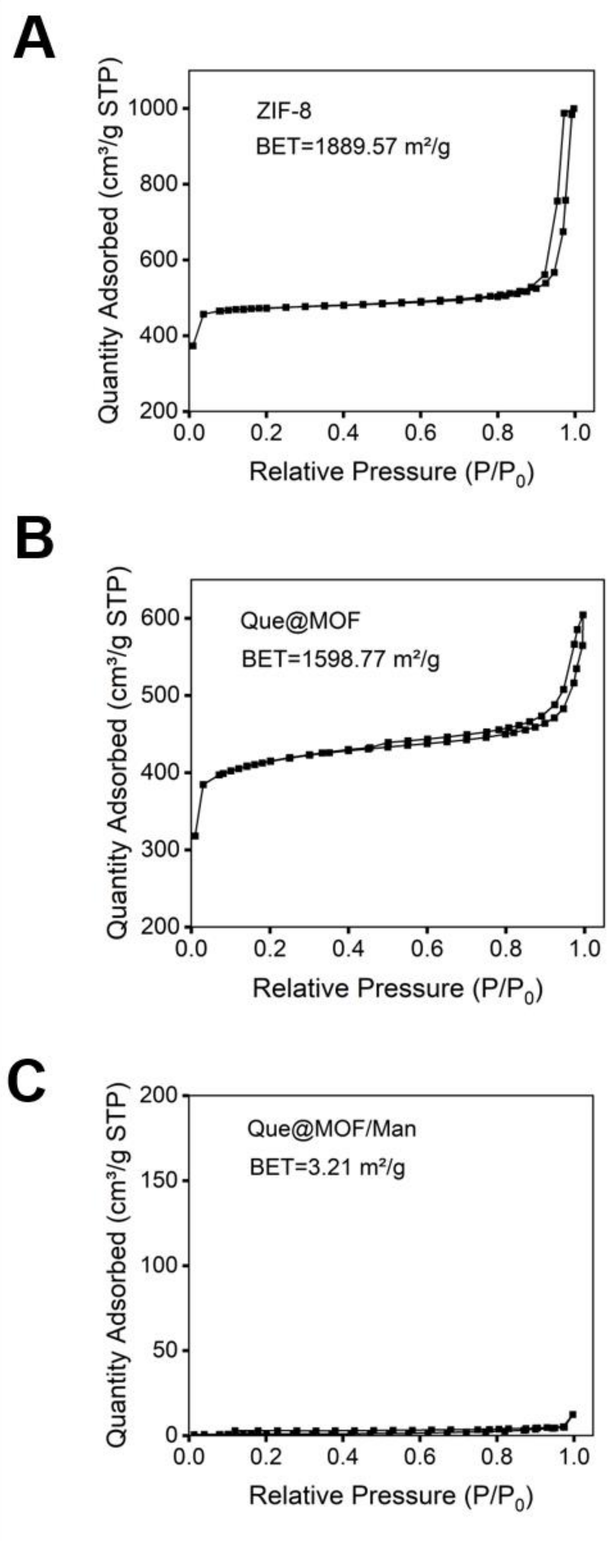

Figure S2. The N_2 adsorption–desorption isotherms of ZIF-8 (A), Que@MOF (B) and Que@MOF/Man (C).

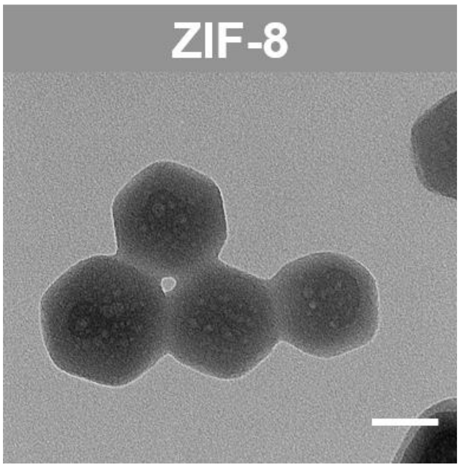

Figure S3. TEM image of ZIF-8. Scale bar, 50 nm.

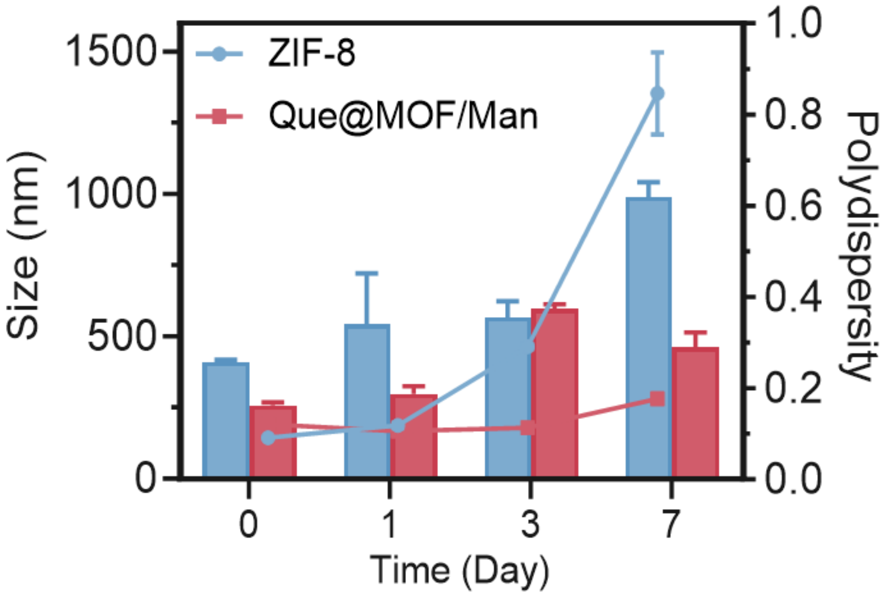

Figure S4. The aqueous stability of ZIF-8 and Que@MOF/Man determined by dynamic laser scattering (DLS) assay.

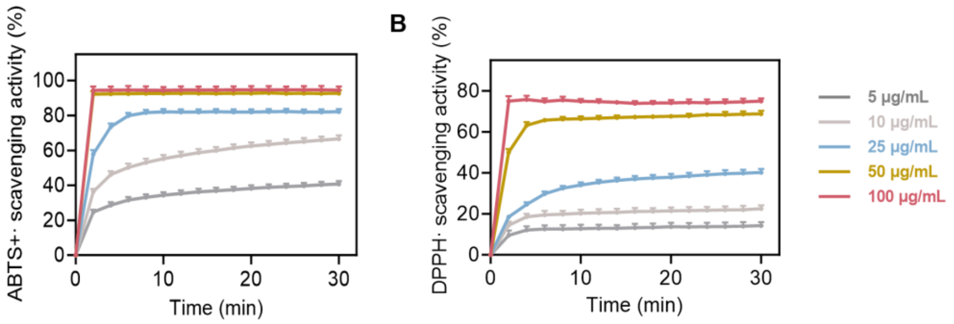

Figure S5. Kinetic curves of the $ABTS^{\bullet+}$ (A) and $DPPH^{\bullet}$ (B) scavenging capacity for various concentrations of Que@MOF/Man in PBS (pH 7.4).

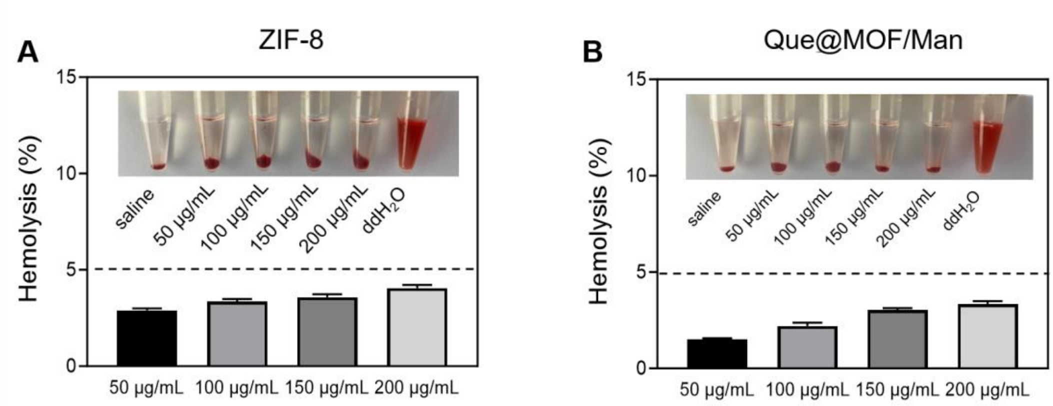

Figure S6. Hemolysis toxicity of ZIF-8 (A) and Que@MOF/Man (B) with different concentrations.

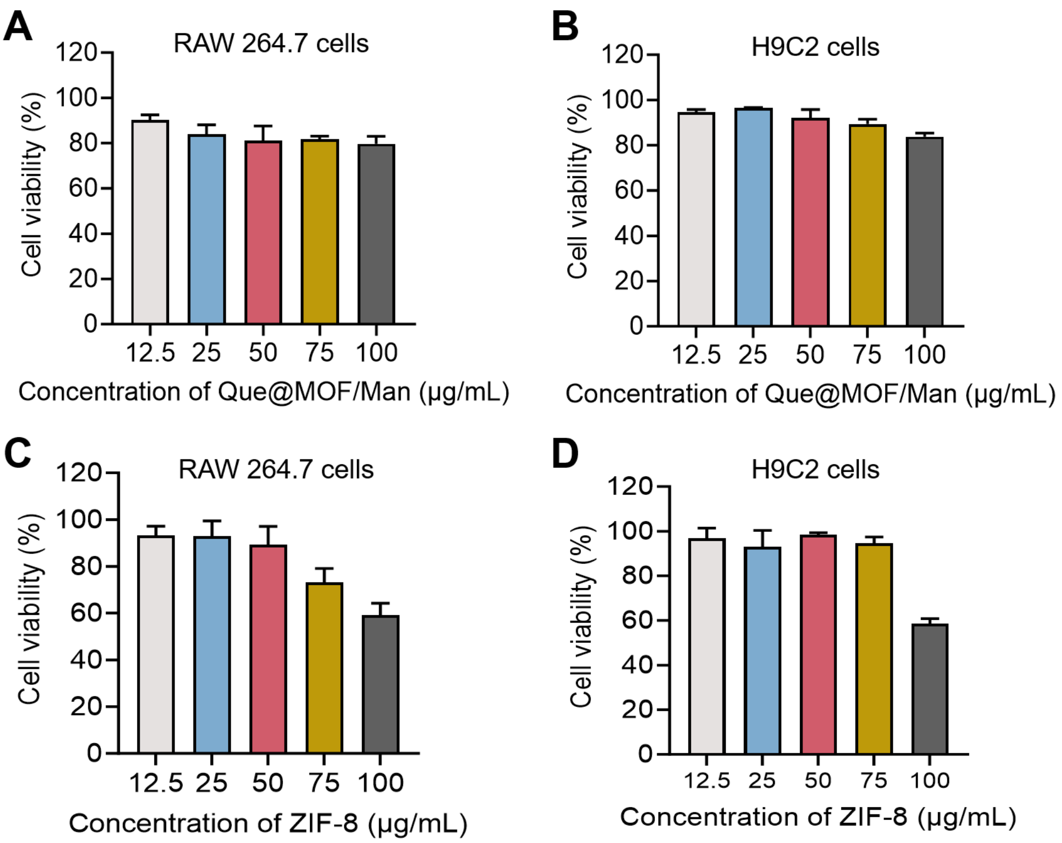

Figure S7. Cell viability of RAW 264.7 cells and H9C2 cells treated with different concentrations of Que@MOF/Man and ZIF-8 nanoparticles.

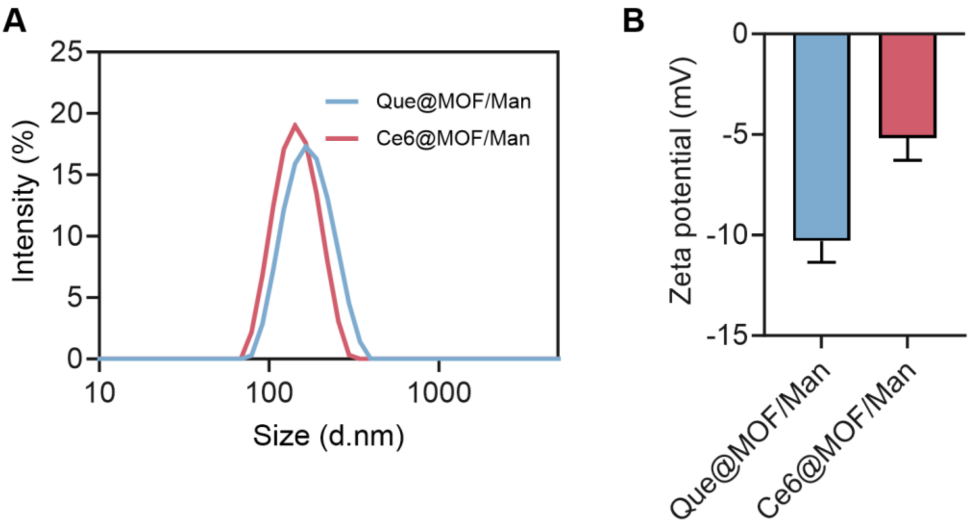

Figure S8. Zeta potential (A) and particle size (B) for Que@MOF/Man and Ce6@MOF/Man by DLS.

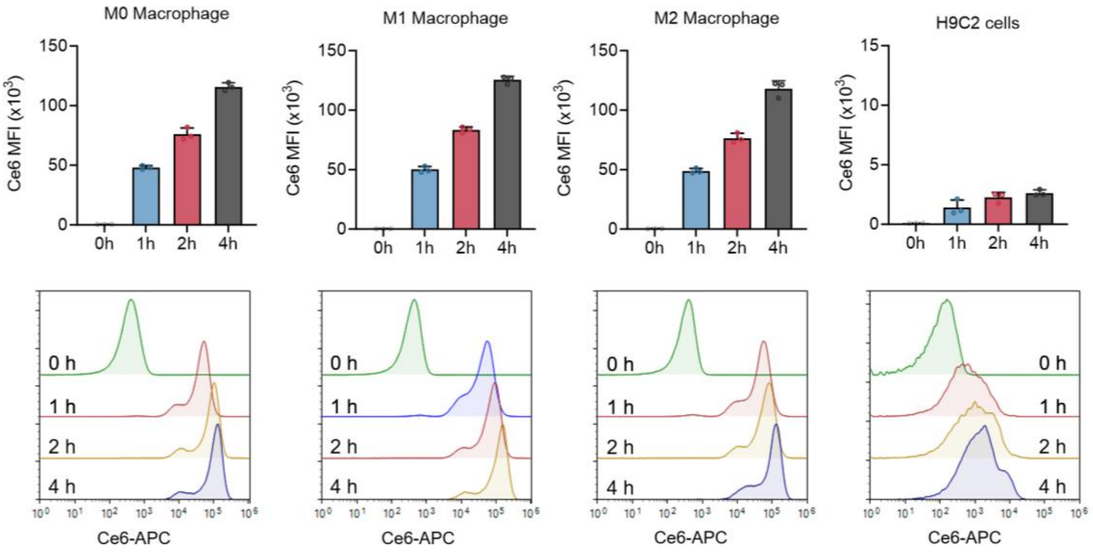

Figure S9. Intracellular uptake of Ce6@MOF/Man in RAW 264.7 cells and H9C2 cells with various incubation time (1 h, 2 h and 4 h) via flow cytometry analysis.

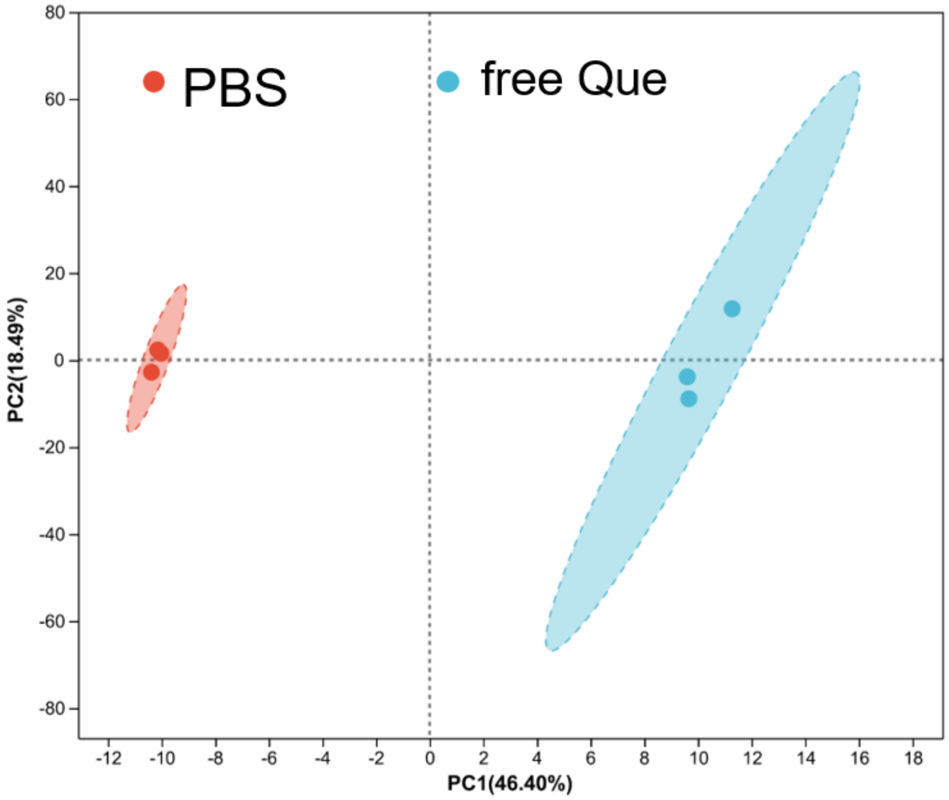

Figure S10. Principal component analysis (PCA) of expressed genes of LPS-induced RAW 264.7 cells after free Que and PBS treatments.

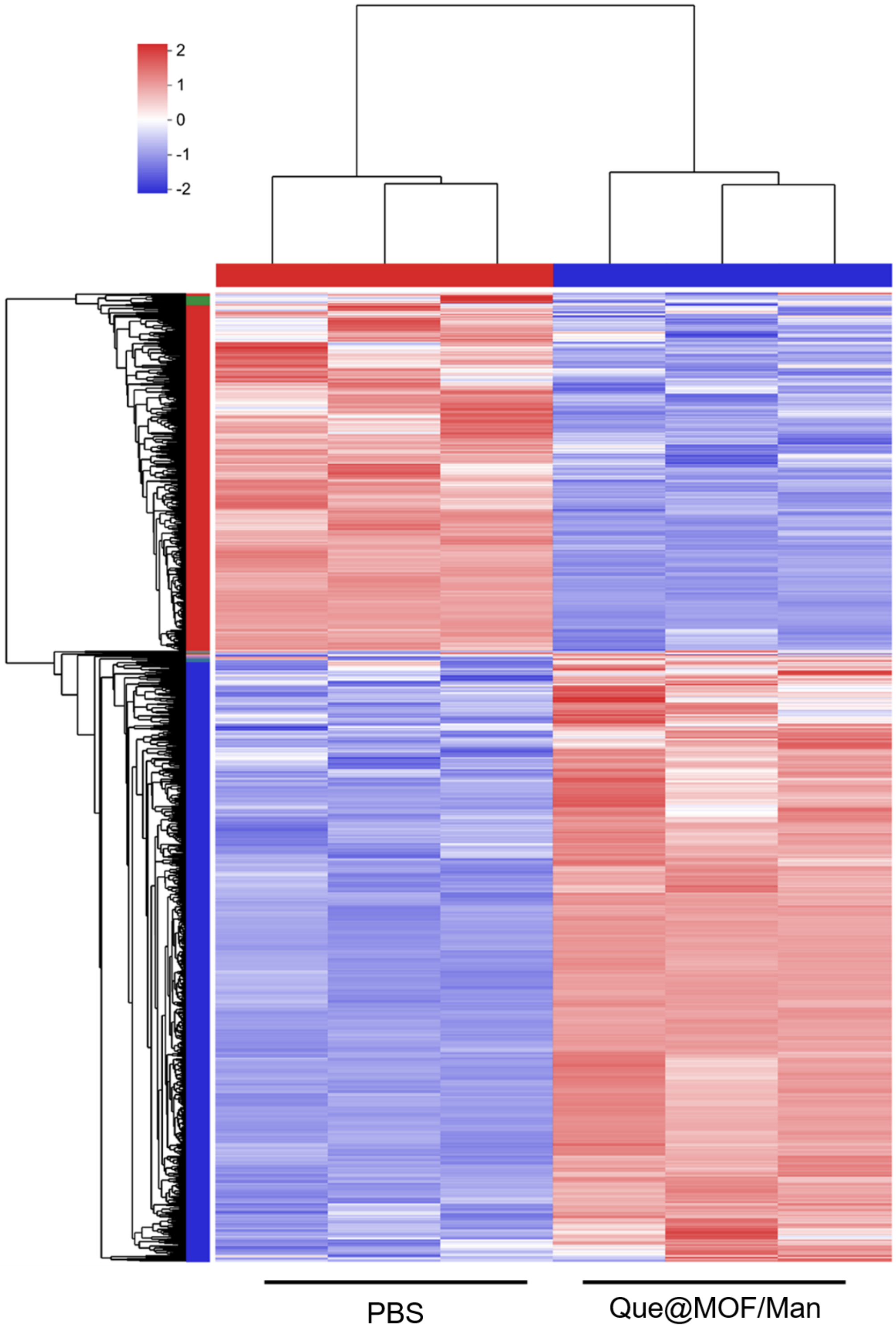

Figure S11. Heat map of differential expression genes (DEGs) between PBS and Que@MOF/Man group.

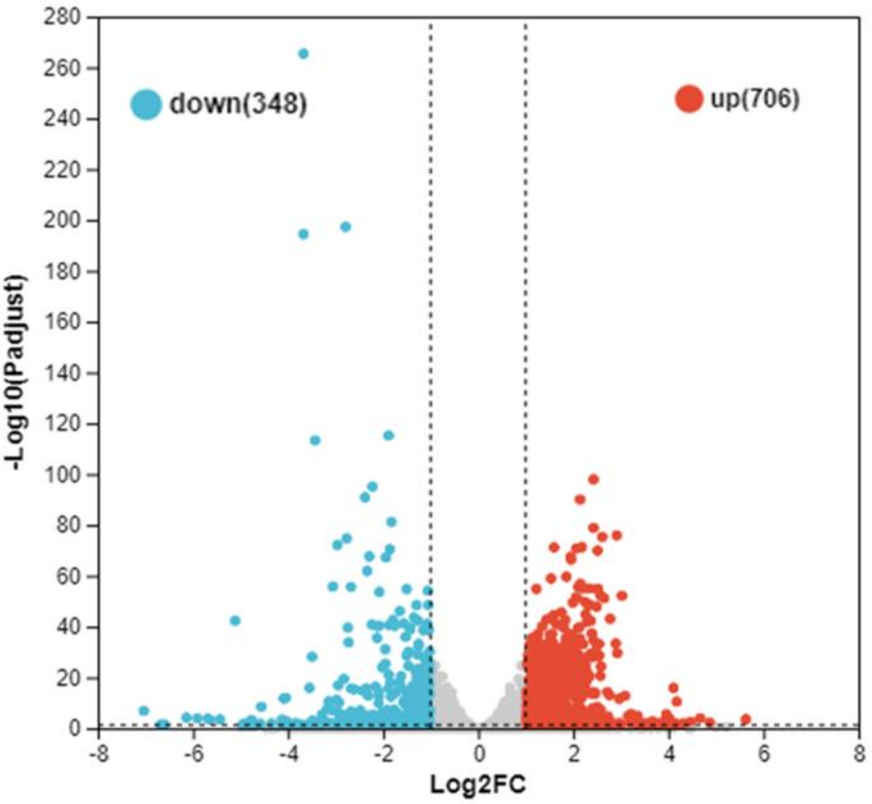

Figure S12. Volcano plot of differential expression genes (DEGs) between PBS and free Que group.

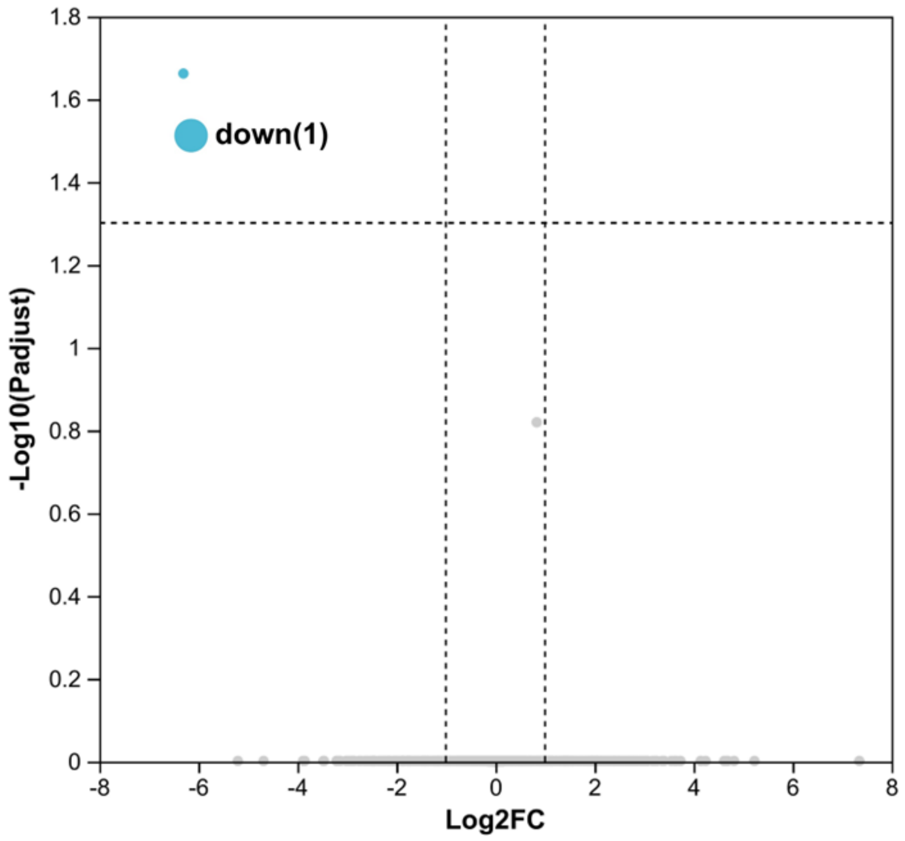

Figure S13. Volcano plot of differential expression genes (DEGs) between free Que and Que@MOF/Man group.

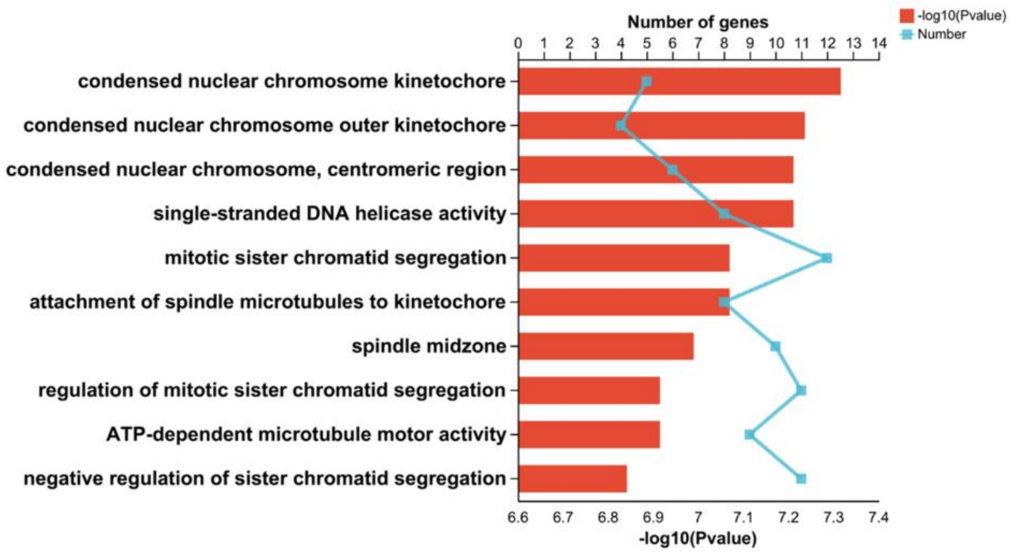

Figure S14. Gene Ontology (GO) enrichment analysis of up-regulated differential expression genes (DEGs) between PBS and free Que group.

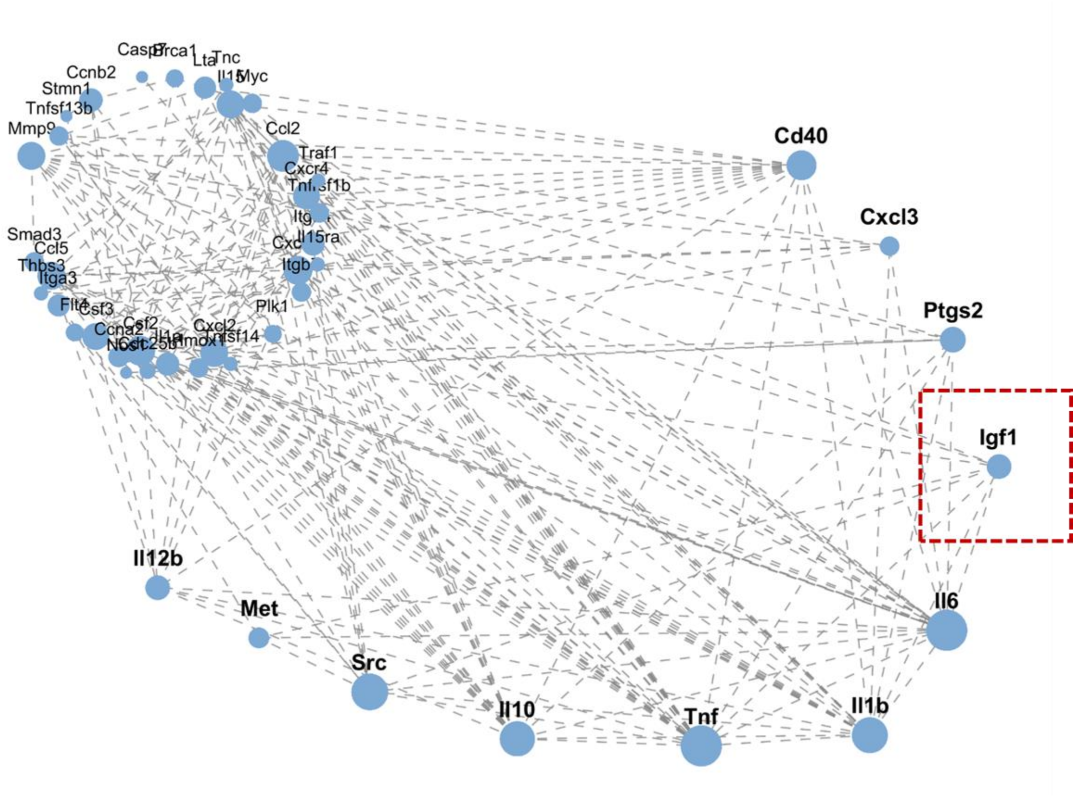

Figure S15. The network consists of proteins encoded by representative differential expression genes (DEGs).

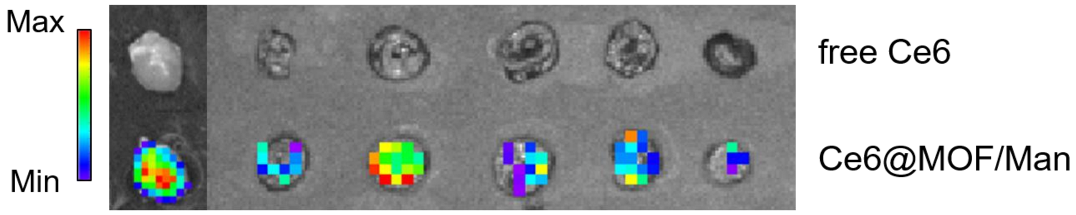

Figure S16. Representative ex vivo fluorescent imaging of myocardium slices 2 h after intravenous injection of free Ce6 and Ce6@MOF/Man.

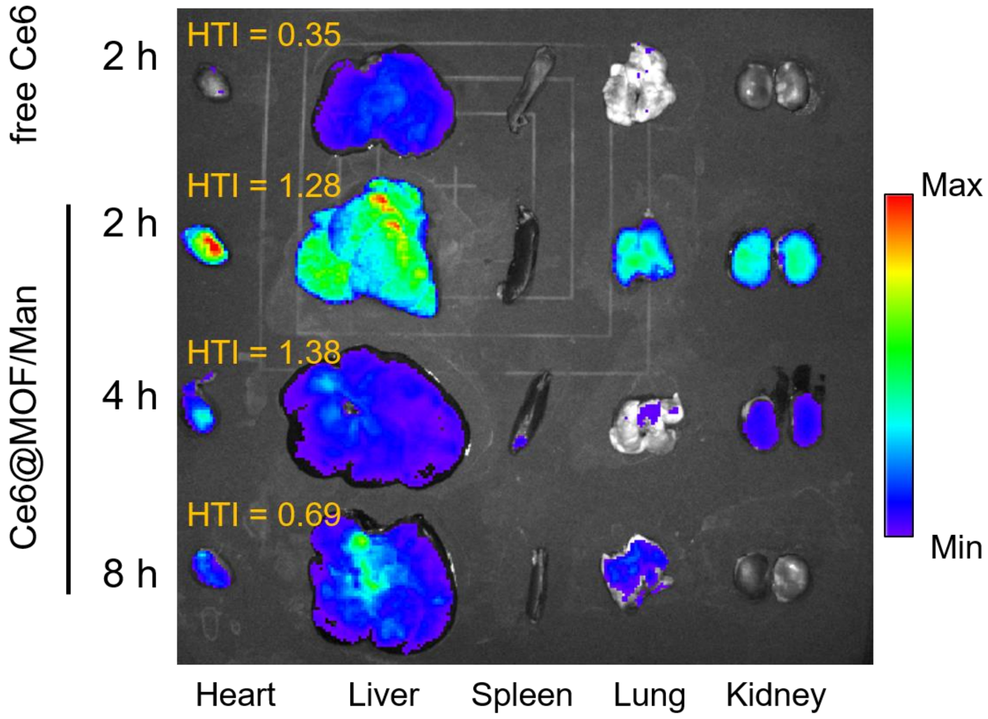

Figure S17. Representative ex vivo fluorescent imaging of major organs (heart, liver, spleen, lung and kidney) at different timepoints (2 h, 4 h and 8 h) after intravenous injection of free Ce6 and Ce6@MOF/Man.

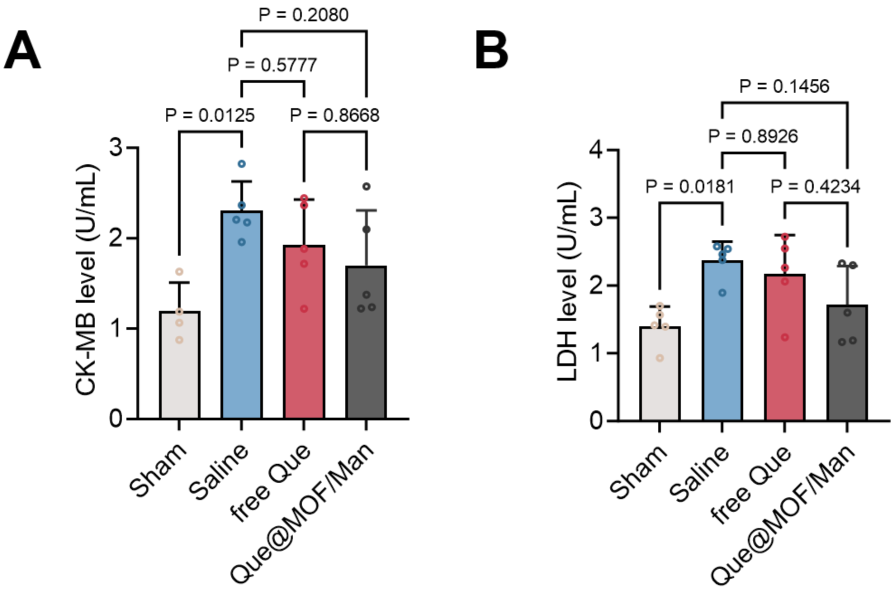

Figure S18. The CK-MB (A) and LDH (B) level in the serum of rats in each group at 3 days after treatment.

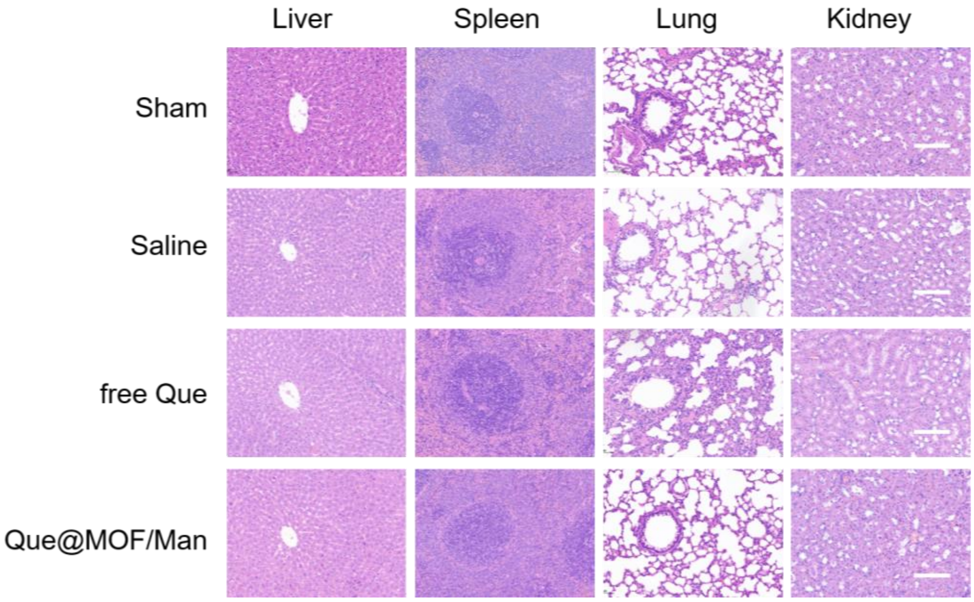

Figure S19. Hematoxylin and eosin (H&E) staining of the major organs (liver, spleen, lung and kidney) in each group at 28 days post-MI. Scale bar, 100 μm.

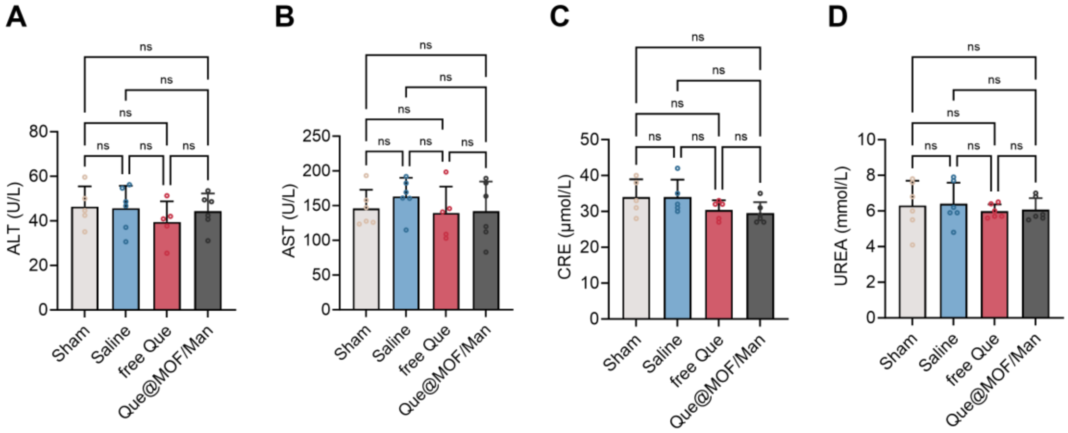

Figure S20. Serum biochemistry analysis of alanine aminotransferase (ALT) and aspartate aminotransferase (AST), and creatinine (CRE) and urea nitrogen (UREA) indicators in each group at 28 days post-MI (n=5-6). ns: no significance.
